# A method to identify and analyze biological programs through automated reasoning

**DOI:** 10.1038/npjsba.2016.10

**Published:** 2016-07-07

**Authors:** Boyan Yordanov, Sara-Jane Dunn, Hillel Kugler, Austin Smith, Graziano Martello, Stephen Emmott

**Affiliations:** 1 Biological Computation, Microsoft Research, Cambridge, UK; 2 Faculty of Engineering, Bar-Ilan University, Ramat Gan, Israel; 3 Wellcome Trust Medical Research Council Cambridge Stem Cell Institute, University of Cambridge, Cambridge, UK; 4 Department of Biochemistry, University of Cambridge, Cambridge, UK; 5 Department of Molecular Medicine, University of Padua, Padua, Italy; 6 Faculty of Engineering Science, University College London, London, UK

## Abstract

Predictive biology is elusive because rigorous, data-constrained, mechanistic models of complex biological systems are difficult to derive and validate. Current approaches tend to construct and examine static interaction network models, which are descriptively rich, but often lack explanatory and predictive power, or dynamic models that can be simulated to reproduce known behavior. However, in such approaches implicit assumptions are introduced as typically only one mechanism is considered, and exhaustively investigating all scenarios is impractical using simulation. To address these limitations, we present a methodology based on automated formal reasoning, which permits the synthesis and analysis of the complete set of logical models consistent with experimental observations. We test hypotheses against all candidate models, and remove the need for simulation by characterizing and simultaneously analyzing all mechanistic explanations of observed behavior. Our methodology transforms knowledge of complex biological processes from sets of possible interactions and experimental observations to precise, predictive biological programs governing cell function.

## Introduction

A major challenge in biology is to move from descriptive narratives towards predictive explanations of biological mechanisms and processes. Interaction network diagrams, now used widely to represent biological systems by mapping components (e.g., genes and proteins) and the possible molecular interactions between them, are a prime example of this challenge. In the absence of an accompanying hypothesis of dynamics and information flow, these maps provide a rich description of the complexity of biological systems, but usually do not confer any explanatory or predictive power.^1^

In an effort to address such shortcomings, both continuous and discrete mathematical approaches have been applied to capture and investigate the dynamics of interaction networks (see ref. 2 for a review). In particular, qualitative (logical) models are a powerful intuitive tool,^1,3^ where the connectivity of a set of components represents excitatory or inhibitory molecular interactions, and logical update functions abstract the involved regulation mechanisms. This allows the dynamical behavior of the system to be studied without the need for detailed biochemical descriptions, which require hard-to-measure kinetic parameters (e.g., synthesis and degradation rates), making the logical modeling formalism an attractive alternative to continuous models.

Logical models are typically constructed through a combination of manual effort and computational techniques,^4,5^ and their dynamics explored by computational simulation or state-space exploration. This can reveal whether the model reproduces known behavior. Model refinement proceeds when simulated behavior is inconsistent with experiment, though this remains challenging for complex networks, as it is non-trivial to infer interactions or update functions manually. Besides the challenge of constructing and refining a suitable model, these approaches introduce implicit assumptions by considering only one of the many mechanisms consistent with observed behavior.^6^ Furthermore, simulation restricts investigation to a limited set of scenarios (e.g., trajectories originating from different initial conditions corresponding to distinct expression profiles), while a complete state-space exploration becomes infeasible as models increase in size.

To address the limitations of such existing approaches, we have developed a methodology that uses automated reasoning (proving the properties of logical formulae using automated algorithms) to transform a description of the critical components, possible interactions and hypothesized regulation rules of a biological process into a dynamic, mechanistic explanation of experimentally observed behavior. Our computational approach allows a large number of possible mechanistic hypotheses and experimental results to be considered simultaneously. Furthermore, it permits experimentally testable predictions of biological behavior to be made that have yet to be experimentally observed, based on all mechanisms consistent with experimental evidence, limiting the bias and implicit assumptions introduced when considering only a single model.

We applied this methodology to the analysis of mouse embryonic stem cell (mESC) self-renewal to derive a highly predictive explanation of known behavior based on simple regulation rules and an unexpectedly small number of key components and interactions, compared with vast interactome diagrams.^7^ The results from applying our approach indicated that the most parsimonious explanation of complex biological behavior can be understood not in terms of prevailing descriptions of a static network, but in terms of a precise, molecular program governing cellular decision making: a minimal set of functional components, interconnected with and regulating each other according to rules that confer to the system the capacity to process input stimuli to compute and output a biological function reliably and robustly.

We propose that a rigorous, formal definition and representation (model) of a biological program, which captures dynamic information-processing steps over time while recapitulating observed biological behavior, is better suited for explaining and predicting cellular (or bio-molecular) processes compared with vast but static interaction network diagrams. Despite the recent progress in studying dynamic interaction networks,^8–14^ a complete framework for the definition, synthesis and analysis of biological programs is missing. Our methodology is designed to identify and analyze such programs, thus advancing the field not only beyond existing techniques, but also beyond prevailing paradigms of thinking in biological science.

Here for the first time, we present our methodology and its theoretical basis, to allow domain experts to apply the technique to their systems of study. We consider three distinct biological systems, and through comparison with studies that utilize existing analysis methodologies, we show how our approach forces us to draw new conclusions to those of the original investigators. For the cell cycle in budding yeast,^11^ our analysis procedures allow us to examine network robustness while avoiding exhaustive simulation sweeps, as well as to establish the requirement for certain interactions, and to predict how the cell cycle is disrupted by genetic perturbations; for myeloid progenitor differentiation,^12^ we predict the requirement for interactions and input signals not previously considered; and for cardiac development,^15^ we predict critical interactions omitted from current models and validate these predictions using results from the literature.

## Results

### Methodology

**Figure 1:**
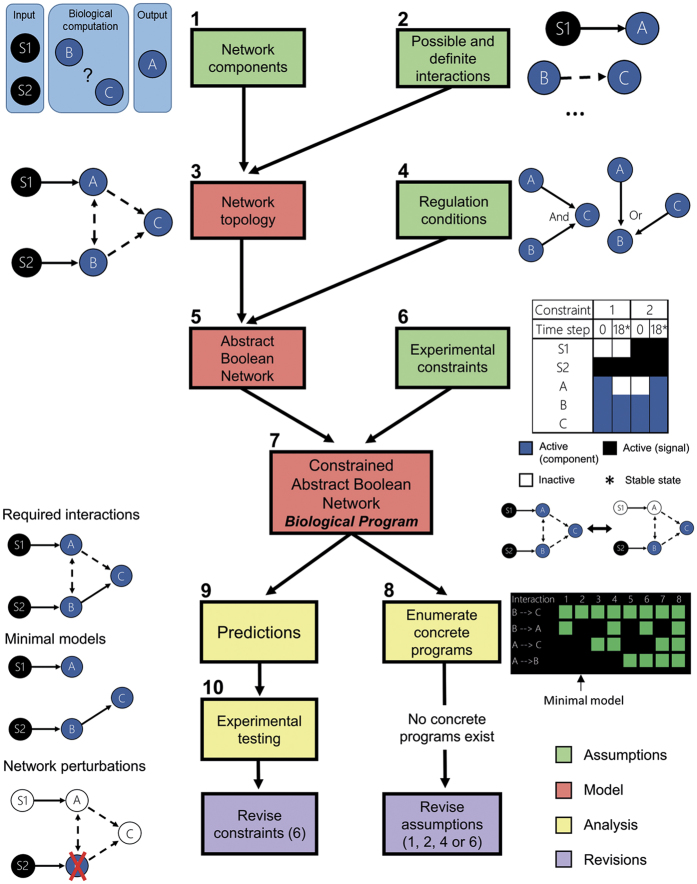
The RE:IN (Reasoning Engine for Interaction Networks) methodology, illustrated by example. First, critical network components must be identified: genes A, B and C are critical regulators of a given cell state, while S1 and S2 are input signals (panel 1). Components can be active or inactive, to fit a Boolean formalism. Second, definite and possible interactions should be defined (panel 2): S1 activates A (solid arrow), B *may* activate C (dashed arrow). These define the topology of an abstract network, which describes 2^4^=16 unique, concrete networks, in which each possible interaction is present or not (panel 3). By combining this topology with known or hypothesized regulation conditions at each node (panel 4), we characterize an Abstract Boolean Network (ABN, panel 5). Next, experimental observations are encoded as constraints on state trajectories (panel 6). A constrained Abstract Boolean Network (cABN) defines an ABN together with the constraints describing system observations, thus integrating available knowledge describing the structure, dynamics and observed behavior of the process (panel 7). We can enumerate the concrete models that satisfy these constraints (panel 8). In addition, we can use the cABN to formulate predictions (panel 9): to identify minimal networks, which have the fewest optional interactions instantiated (concrete model 2, panel 8), as well as required (or disallowed) interactions that are present in all (none) concrete models. We can also study genetic perturbations. Once predictions have been tested experimentally (panel 10), they can be added to the set of experimental constraints. If no concrete models are identified, then the process is iterated, starting by re-examining our assumptions about components, interactions, dynamics and behavior.

**Figure 2:**
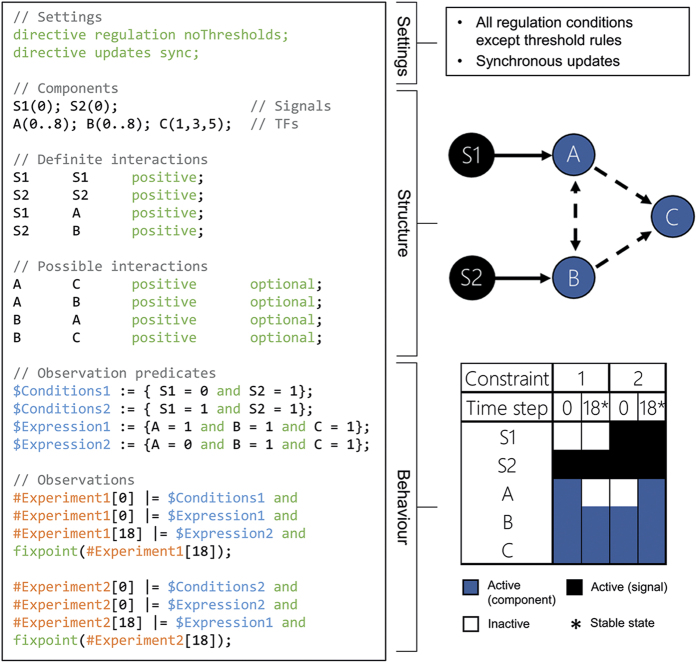
Encoding sets of models and constraints in RE:IN. Shown here is how to encode a set of components, regulation conditions, interactions and constraints in RE:IN, using the toy example from Figure 1 as an illustration. This highlights how to set assumptions, such as a synchronous update scheme, whether to include the Threshold regulation conditions, and how to restrict the set of regulation conditions for a specific components (e.g., C can only use conditions 1, 3, or 5). Constraints are defined as individual experiments, in which component states are defined (using labelled predicates, if desired) at specified time points. We also highlight how to define such a state to be a fixed point.

We use a simple, demonstrative example, summarized in Figure 1, to provide an overview of our methodology. We illustrate the approach and assumptions inherent in the construction of a set of logical network models from experimental data to describe a process of interest, and the subsequent analysis that can be performed. In Figure 2, we present the encoding of this simple model and assumptions as an illustration of the intuitive domain-specific language we propose, while formal definitions of the concepts described are provided in Materials and Methods.

First, the input and critical components of the biological process must be defined, together with its output, which represents the biological decision to be explained (Figure 1, panel 1). Inputs can be chemical signals, mechanical triggers or signaling cascades, and the output could represent a cellular decision or phenotype: e.g., whether a stem cell differentiates or remains pluripotent,^7^ which cell type to differentiate into,^12,15^ or whether to undergo division.^11^ This can be captured by the state of the network components.

When selecting the initial set of critical components to include, they should be functionally relevant: only those that have a substantial effect on the process under study when inactivated or overactivated. Various combinations of genes, proteins, protein complexes, non-coding RNAs, metabolites and signaling molecules can be considered, identified by literature search or genetic screens. This set can be revised if model refinement is required (see below).

Within logical modeling, variables take a discrete number of states. Here we abstract the activity of each component to two possible states: ON, representing a gene that is actively expressed at endogenous levels, a transcription factor (TF) present in high enough concentrations to be functional, or a protein in its active conformational form, and OFF otherwise. While gene regulation and signaling pathways are not always digital, they have been successfully treated as Boolean values in several instances, e.g. as markers of cellular states or genes active during specific phases of the cell cycle.^16,17^

Second, potential interactions between components should be identified, which must have both sign (positive or negative, for activation or inhibition, respectively) and direction (panel 2). An interaction could represent the direct binding of a TF (source) to the promoter of a downstream gene (target) or a post-transcriptional modification of the gene’s product, and can be inferred from a range of data types (Table 1 and Supplementary Material). Interactions may also represent indirect effects, in the case where a secondary regulatory effect has been captured by the data.

Interactions are classed either as definite, if supported by multiple sets of reliable experimental evidence, or possible, to indicate the option of a putative interaction. For example, for transcriptional regulation, it is generally accepted that measuring gene expression shortly after a genetic or chemical perturbation allows secondary effects to be ruled out, but chromatin immunoprecipitation or promoter assays should also be used to further support a direct interaction before labeling it as definite. For post-translation modification, mutagenesis of individual residues and *in vitro* assays are generally accepted as strong evidence for a given interaction. As the absence of an interaction is as strong an assumption as defining one to be definite, possible interactions should be used if there is uncertainty. In the absence of sufficient experimental evidence, it is possible to consider interactions between all components as possible.

Altogether, the set of interactions define an abstract network topology (panel 3), so called because an abstract network with 4 possible interactions generates 2^4^=16 unique, concrete topologies, in which each possible interaction is present or not.

The next step is to augment the static network topology with information that determines transitions between system states, using logical rules that describe how each component updates in response to the state of its regulators (panel 4). We remove the need to specify individual update functions in the network, which are often difficult to elucidate^10^ and, importantly, require knowledge of the exact network topology. Instead, we generated a set of 20 biologically meaningful regulation conditions that are compatible with all topologies defined by the abstract network.^18^ We achieve this by defining rules according to whether none, some or all of a components activators/repressors are present. The complete set of update functions, which are consistent with several assumptions, are defined together with a threshold rule (Materials and Methods).^11^ If prior experimental evidence can eliminate one or more regulation mechanism for a given component, for example that a component requires at least one activator in order to switch on, then a subset of these regulation conditions can be assigned in accordance with model assumptions. The overall network can be updated synchronously (where all components update at each step) or asynchronously (one component per step). As the update functions we consider are deterministic, synchronous updates lead to deterministic behavior, while asynchronous updates lead to non-determinism due to the sequence of component updates.

By defining the set of critical components, possible and definite interactions (the abstract network topology) together with the allowable regulation conditions, we construct an Abstract Boolean Network (ABN): a formal representation that defines the possible structure and dynamics of unique, concrete networks (panel 5). The ABN thus encodes all possible mechanisms that could potentially explain experimental observations. ABNs generalize the concept of Boolean Networks (BNs)^19^ as the state of each component is represented by a Boolean value, but not all interactions and regulation conditions are instantiated. In contrast, a concrete network includes only definite interactions, and a single regulation condition per component, and can be viewed as a BN.

We seek the set of concrete networks from the ABN that are consistent with experimental observations, which are derived both from new data and the literature, and are encoded by specifying the states of some or all of the components along unique trajectories of the system (panel 6). This introduces restrictions on the choice of possible interactions and regulation conditions assigned to each component, to ensure all observations are satisfied. When a network satisfies all observations, as part of the solution, a complete trajectory (where all unknown component states are instantiated) is identified for each constraint as a demonstration and potential explanation of how the expected behavior can be realized.

Observations can describe the change in system behavior under different inputs, or under genetic manipulations, by defining initial and subsequent cellular states. In the simple example in Figure 1, we require all components to be active under both signals, but when only S2 is present, B and C are active, while A is inactive. A state can be defined as stable, such that subsequent updates will not lead to state changes. This provides a mechanism for describing cellular decisions that persist indefinitely, e.g., the stable gene expression pattern observed in a differentiated cell. Alternatively, cycles that follow a sequence of intermediate states can be described, even when the precise time of these states is unknown. In addition, the observed effects of the inactivation or over-activation of a component can be specified. The three case studies we present below illustrate such constraints.

A constrained Abstract Boolean Network (cABN) is the formal representation of the ABN together with the constraints describing the observed behaviors of the system (panel 7). It thus represents all possible mechanisms, i.e., concrete topologies and regulation conditions, consistent with observed system behavior.

The cABN description is grounded in logic and permits the application of automated reasoning. This is a powerful analysis strategy, where valid conclusions are drawn directly from the cABN definition through logical inference and efficient model finding algorithms. We encode this representation as a Satisfiability Modulo Theories (SMT) problem, in which logical expressions are constructed that define the possible combinations of interactions and regulation conditions, and the resulting network behaviors over time. This approach reflects how experimental observations might be interpreted manually given an interaction network diagram (e.g., component A either activates or represses component B; down-regulation of A leads to upregulation of B; therefore, A must repress B). We solve the SMT problem within a bespoke tool: the Reasoning Engine for Interaction Networks (RE:IN), which uses the bit-vector theory reasoning strategies^20,21^ implemented within the SMT solver Z3^22^ (Materials and Methods). RE:IN is made freely available as a cloud-based application (rein.cloudapp.net), with examples and tutorials provided (research.microsoft.com/rein).

The set of consistent networks can be enumerated and examined individually (panel 8) using RE:IN, which also identifies when no such networks exist, prompting us to re-examine our initial assumptions (Figure 1, green boxes). For example, additional possible interactions could be included in the abstract network as part of model refinement. If solutions do exist, then we can impose a limit on the number of possible interactions to consider, which allows us to derive minimal networks that are easy to examine and can reveal components and interactions essential for the biological process. These correspond to one of the simplest explanations—in terms of numbers of interactions—of the behavior the network is expected to produce. Alternative definitions of ‘minimal’ might focus on restricting the number of components, or the possible regulation conditions. In the example, there is one such minimal model, containing only the activation from B to C.

Even without enumeration, we can pose and test various hypotheses to explore whether certain behavior is guaranteed in the system regardless of the precise mechanism, and identify the exact steps that lead to a specific output. This is significant, particularly in cases where the number of concrete networks is too large to be feasibly investigated. We consider all consistent models simultaneously, thereby assuming them to be equally valid and eliminating the bias introduced when only a single model is studied.

First, we can study those interactions critical to the network. Required interactions, if individually excluded, will prevent the constraints from being satisfied. In the example, it is required that B activates C (panel 9). Similarly, interactions that must be disallowed are those that if enforced as definite, would prevent the constraints from being satisfied. Note that if all outgoing interactions from a component are found to be disallowed, this reveals that the component is not required to behave as a regulator, and could be removed from the analysis if there is no additional biological evidence for its importance.

Second, we can formulate predictions by determining whether a new hypothesis, encoded as an additional constraint, is satisfied by the cABN. We guarantee that the prediction is implied by all consistent mechanisms by showing that the converse of this constraint (the null hypothesis) is unsatisfiable. For example, we predict that inactivation of B in the presence of S2 and absence of S1 causes A and C to become inactive (panel 9). Indeed, useful insights are identified even when no prediction can be generated for a given query, as this signifies that some mechanisms support the hypothesis, and other mechanisms support the null hypothesis, suggesting a discriminating biological experiment to refine the set of models further.

Note that, in general, the size (number of concrete models) of the cABN relates to its predictive capacity: increasing the number of possible interactions increases the number of concrete networks that can potentially produce different dynamic behavior, which in general, reduces the number of predictions that can be formulated. Interactions with less experimental support can be included as part of a model refinement process if no consistent models exist.

Following experimental testing of predictions, novel biological knowledge can be incorporated as new experimental constraints (panel 10). Even if a prediction holds true it is recommended to add constraints explicitly capturing these new data before further expanding the cABN.

To illustrate further the application and implementation of our methodology, we consider three separate biological systems, using models from the literature as a concise representation of the domain knowledge of critical components, interactions and behaviors. When starting from experimental data alone, domain experts can apply the workflow from Figure 1 instead. We provide a table summarizing these studies in Supplementary Material.

### Cell cycle regulation in yeast

To study the cell-cycle in budding yeast, Li *et al.*^11^ constructed a synchronous BN of 12 regulators, applying a threshold update function (Materials and Methods) to each component. The network is shown to recapitulate a trajectory through the temporally ordered phases of the cell cycle (without prescribing the exact step at which each phase is reached) upon perturbation of the stationary G1 phase, before returning to this stable state.

**Figure 3:**
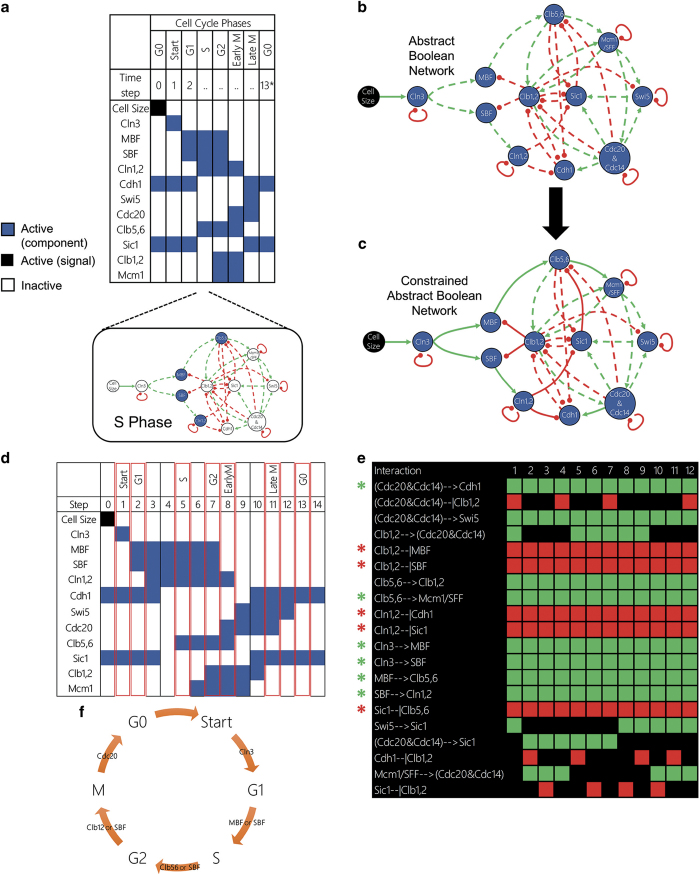
Studying the biological program governing the cell cycle in budding yeast. (**a**) The order of the cell cycle phases upon perturbation of G0 due to activating cell size, before the system stabilizes in G0 (indicated by a star). An example of S phase is visualized graphically on the network diagram. (**b**) The ABN constructed from the Yeast model proposed by Li *et al.* (**c**) The cABN satisfying the cyclic constraint in (**a**). 11 required interactions are indicated by solid arrows (in addition to the definite activation of Cln3 by cell size). (**d**) Example trajectory taken by one solution when the G0 state is perturbed by activating cell size. The step at which each cell cycle phase is reached is indicated. (**e**) There are 12 minimal networks, each consisting of 20 instantiated possible interactions. Green indicates an activation, red indicates a repression, and asterisks indicate required interactions. Some of these mechanisms do not require all components to behave as regulators (Mcm1, Cdh1 and Swi5). In addition, some sets of interactions expose redundancy: for example, six concrete models do not require Swi5 to regulate Sic1, which is instead activated by Cdc20. In the remaining models, Swi5 is required to activate Sic1 in the absence of activation by Cdc20. (Similarly, the activation of Cdc20 by Clb12 or Mcm1, and the inhibition of Clb12 by Cdc20, Cdh1 or Sic1.) (**f**) The set of consistent mechanisms can be used to predict perturbations that arrest the cell cycle. In each case, loss of function of the gene highlighted on the arrow will prevent the transition from occurring.

Encoding this concrete model in RE:IN confirms that it satisfies the cyclic constraint (Figure 3a). However, by instead marking the set of interactions as possible, we can quickly examine the robustness of the network (Figure 3b). The maximum number of models that could potentially satisfy the constraint is 2^29^=536, 870,912. By enumeration with RE:IN, we identified 4,480 consistent mechanisms, demonstrating that it is possible to remove interactions from the concrete network without compromising expected behavior. To infer this by simulation alone would require exhaustive, time-consuming trajectory sweeps.

Furthermore, we investigated which interactions are required to satisfy this constraint; a question that cannot easily be asked of a single, defined network. We identified that 11 of the possible interactions are required (Figure 3c), which we predict must be present in any valid explanation of the cell cycle, assuming the initial set of interactions shown in Figure 3b. An example trajectory for a single concrete network that illustrates the cycle is shown in Figure 3d. Further, we identified 12 minimal networks, each with 16 instantiated possible interactions (Figure 3e). Upon examination, these expose the redundancy of including both a direct and indirect interaction between two genes in the original BN, e.g., Cdc20 activating Sic1 directly, and indirectly through Swi5. Three components are not required to act as regulators in some of the minimal networks (Mcm1, Cdh1 and Swi5), and therefore could be removed from these specific models without affecting the dynamics of the remaining components. This illustrates the usefulness of minimal networks to investigate how to reduce the number of components considered, in addition to the number of interactions.

We also investigated the consequence of gene inactivation on cell cycle progression, testing whether the set of consistent models can complete the transitions between the cell cycle phases under perturbation. This allowed us to predict genes essential for cell cycle progression, and where the cycle might arrest. We predict at least one gene inactivation that will arrest each phase transition (Figure 3f). All but one of these predictions are consistent with the literature, in which arrest or delay in cell cycle progression arises following inactivation of these genes (Table 2). To conduct model refinement, the prediction to be corrected can be added to the set of constraints using the information derived from the experimental test. Given it will not be possible to satisfy this new constraint with the current set of assumptions, these should next be revised, for example, by including additional possible interactions (Figure 1).

Here we have demonstrated that alternative, simpler mechanisms are capable of producing the expected behavior of the cell cycle in budding yeast, and by encoding the model as a cABN, that it is robust to adaptations (Figure 3c). This demonstrates how to achieve an understanding of the system while avoiding the need for simulation or exhaustive enumeration of trajectories by reasoning about the behavior of all consistent networks, and how to formulate predictions of genetic perturbations.

### Myeloid progenitor differentiation

**Figure 4:**
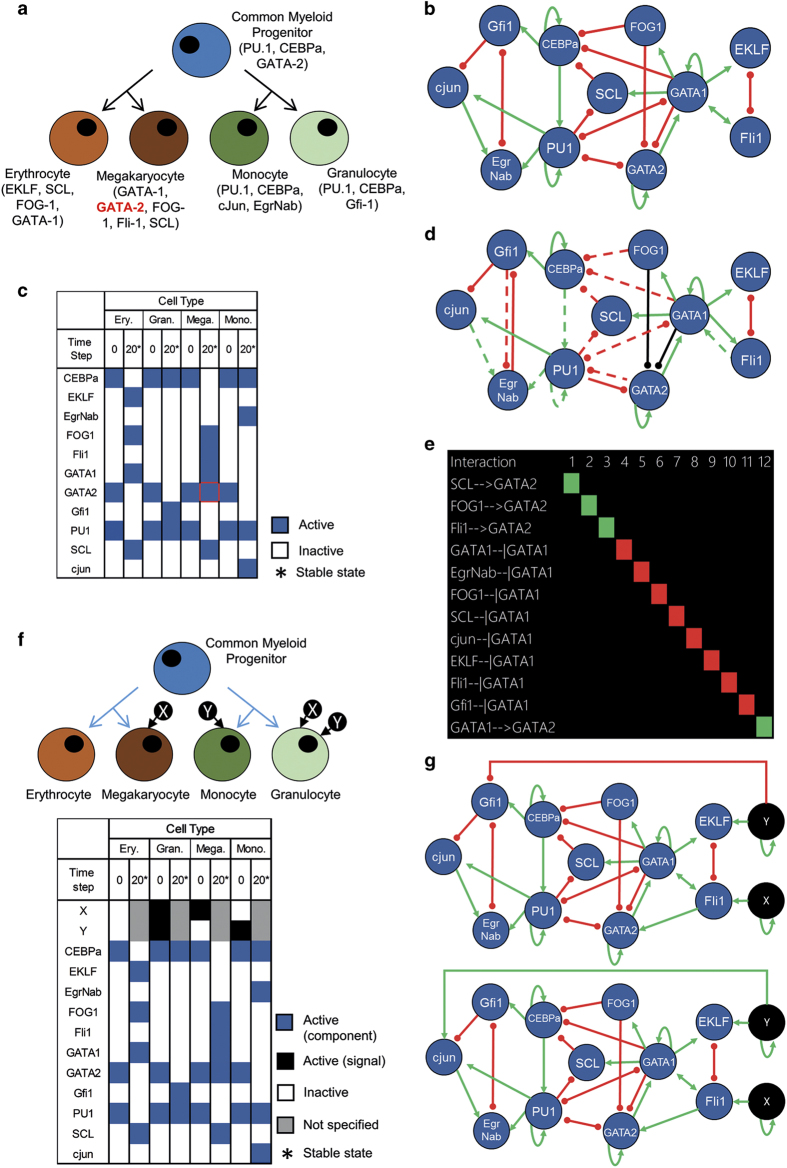
Studying the biological program governing myeloid progenitor differentiation. (**a**) The differentiation of a common myeloid progenitor towards four different blood cell types is considered. (**b**) The network topology proposed by Krumsiek *et al.* (**c**) The set of experimental observations indicates that, starting from the progenitor cellular state (step 0), each state characterizing a different cell type is reached after 20 steps and the system stabilizes (indicated by a star). The megakaryocyte GATA-2 was observed as active in experiments but was inactive in the model from Krumsiek *et al.* (red box). (**d**) 15 of the possible interactions were identified as required (solid red and green arrows) and 2 were identified as disallowed (solid black arrows) in the cABN satisfying the constraints in **c**. (**e**) If all interactions from the original model in **b** are considered as definite, the correct expression of megakaryocyte GATA-2 can be achieved by including one of 12 possible interactions. (**f**) The experimental constraints are modified to specify that the cell-fate decision is made in response to whether the hypothetical signals X and Y are present or not. (**g**) Two minimal models are identified when considering the hypothetical signals. Three novel interactions (signal X activating Fli1, signal Y activating EKLF and Fli1 activating GATA-2) appear in both models. In the first minimal model Y represses Gfi1, while in the second this signal activates cjun.

To model myeloid progenitor differentiation (Figure 4a), Krumsiek *et al*.^12^ constructed an asynchronous BN of 11 regulators and 28 interactions based on the literature (Figure 4b). By directly exploring the 2^11^=2,048 nodes of the state-transition graph, four stable states (attractors) were shown to be reachable from a common progenitor state. The gene expression pattern characterizing each attractor was shown to correlate with messenger RNA expression data obtained from erythrocyte, megakaryocyte, monocyte and granulocyte cells, with the exception of GATA-2 in megakaryocytes, which was defined as inactive in the model but observed experimentally as highly expressed.

We first studied this proposed network topology (Figure 4b). The specified update functions named regulators for each component, and so we instead applied our regulation conditions, assuming at least one activator is required for component activation (Figure 2). We employed an asynchronous update strategy, and used the gene expression patterns of the 5 cell types as observations (Figures 4a and c). RE:IN identified that these constraints are satisfiable, despite our use of potentially different regulation rules. Interestingly, no solutions were found using only the threshold rules, indicating that additional regulation conditions, for example those we propose, are required.

If we correct the constraint that GATA-2 is active in megakaryocytes, as observed experimentally,^12^ no consistent models exist. This is not the case if every interaction is marked as possible, and under this scenario we identified that to reproduce the observed behavior, 15 interactions are required and 2 are disallowed (Figure 4d). However, previous experimental evidence supports the inclusion of these two disallowed interactions.^12^

An alternative strategy for satisfying observed behavior is to assume that all interactions from the original model have been validated, but additional interactions are missing. To investigate this, we constructed an ABN by setting the interactions from Krumsiek *et al.* as definite and adding all other interactions (activation and repression between each pair of components) as possible. Identifying the minimal networks in this case reveals that the observations can be reproduced with only one additional interaction (Figure 4e). Our results suggest 12 candidate interactions, at least 3 of which (Fli1 to GATA-2, SCL to GATA-2, Gfi1 to GATA-1) are consistent with interactions reported elsewhere.^23,24^

Krumsiek *et al.* assumed that the precise order in which genes are updated determines the differentiation of a progenitor cell into one of four cell types. An alternative approach, consistent with our view of biological programs, would be to describe this decision as the result of the deterministic information processing of a number of inputs (e.g., cytokines) that regulate haematopoiesis.^25^ To illustrate this, we considered two hypothetical signals (X and Y) that deterministically specify cell fate (Figure 4f), and employed synchronous updates. Once set, the signals remain unchanged, but their effects can propagate throughout the network over a number of updates. With no prior knowledge of how such signals could input to the network, we included a possible positive and negative interaction from each signal to every component of the network, while again considering all original interactions as definite, and the 12 interactions from Figure 4e as possible. We then identified that there are only two minimal models (Figure 4g). In both, Fli1 activates GATA-2, and signals X and Y activate Fli1 and EKLF, respectively. The two mechanisms differ only in whether Y activates cjun, or represses Gfi1.

Here we have shown how our methodology can be applied to search for additional interactions, and that non-deterministic updates can be replaced by a deterministic biological program with precisely defined inputs. We employ minimal networks to reveal candidate signal targets.

### The murine cardiac gene regulatory network

**Figure 5:**
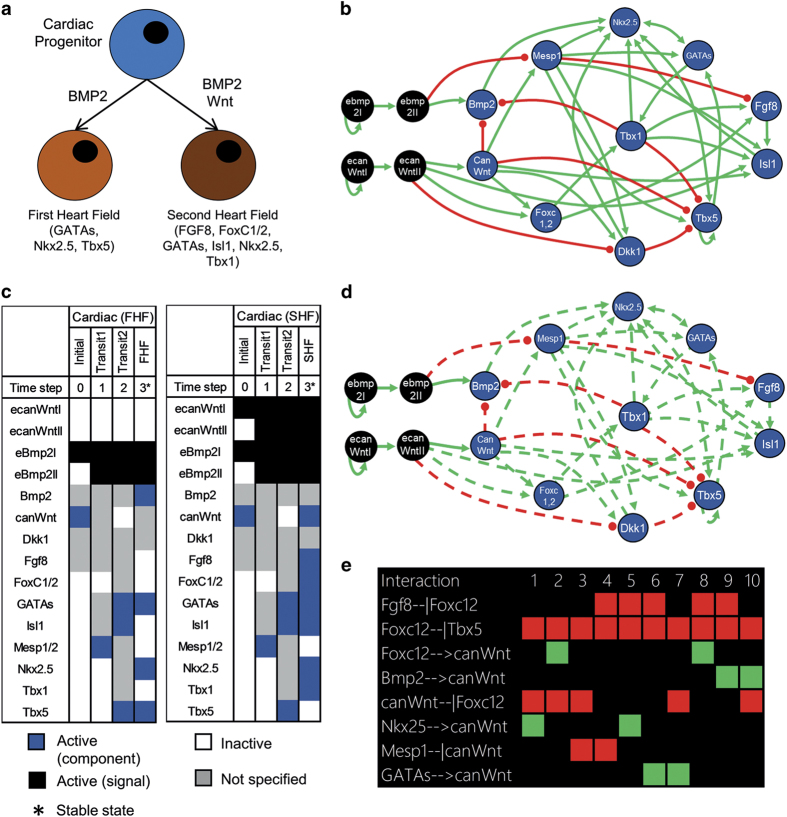
Studying the biological program governing cardiac development. (**a**) The differentiation of a cardiac progenitor cell towards either the first or second heart field as determined by Bmp2 and canonical Wnt signaling. (**b**) The ABN constructed based on cardiac model proposed by Herrmann *et al*., with Bmp2 and canonical Wnt signaling represented using two nodes to model a time delay. (**c**) The set of experimental constraints that the cardiac system exhibits. The initial and stable final expression states are shown, together with the expected temporal dynamics. (**d**) The ABN with all interactions set as possible. (**e**) The 10 minimal models that can satisfy all constraints, each of which contains an additional three interactions to the set defined by Herrmann *et al.*

At the end of gastrulation, a developmental decision occurs when the cardiac mesoderm splits into progenitors of the first and second heart field (FHF/SHF; Figure 5a). To model heart development in the murine embryo, Herrmann *et al.*^15^ constructed a synchronous BN composed of 11 key regulators with two input signals corresponding to Bmp2 and canonical Wnt signaling, based on published data (Figure 5b), which they investigated by simulation. They also presented expected gene expression states along the transition to either FHF or SHF (Figure 5c).

By encoding their concrete BN in RE:IN, we found that while it is consistent with the stable, final gene expression patterns for the FHF and SHF, it cannot satisfy the expected temporal dynamics throughout the transition (Figure 5c). Indeed, removing any interactions from the cABN does not make this constraint satisfiable, which we easily examined by setting all interactions as possible, instead of definite (Figure 5d).

To identify new potential interactions to resolve this inconsistency, we included all positive and negative interactions between the eleven components that were not included in the original BN as possible, while keeping the original interactions as definite. This assumes sufficient experimental evidence for the interactions identified by Herrmann *et al.* Encoding this larger ABN with the experimental constraints in RE:IN identified a consistent set of concrete mechanisms. Moreover, only 10 minimal networks exist, which each require the addition of 3 out of 8 new interactions (Figure 5e). There is evidence for 6 out of the 8 new interactions in the literature (Table 3,^26–32^), which suggests that our approach led to the identification of plausible missing connections in the program governing cardiac development.

### Comparison with alternative approaches

We compared our methodology against two alternative approaches: a naive brute-force simulation strategy, and the Cell ASP Optimized (caspo) tool,^33^ based on Answer Set Programming (ASP). The ASP approach focuses on optimization, and attempts to find the set of minimal networks that best reproduce observed behavior, with a tolerance parameter controlling network size that can be adjusted to generate sub-optimal solutions. Further details of this comparison are presented in Supplementary Material.

For the simple cABN shown in Figure 1, the simulation approach searched through all 3,888 concrete models (unique in interactions and regulation conditions) in ~2 min, to identify the 1,080 consistent models. In contrast, RE:IN enumerated these 1,080 solutions in about 15 s. Focusing on unique topologies only, caspo identified 6 valid, sub-optimal concrete networks, while RE:IN identified 8 (Figure 1, panel 8). Both tools performed this analysis in under 1 s, and consistently identified the required activation of C by B. Furthermore, caspo identified the required activation of A by S1 and B by S2, while these interactions were set as definite using RE:IN (Figure 1). Interestingly, the 2 additional solutions identified by RE:IN involve a feedback loop between components A and B. Lastly, both tools identified the single minimal model in under 1 second.

Next, we considered deterministic myeloid differentiation with signals X and Y (Figure 4f). Analysis using caspo led to memory errors, potentially caused by the complexity of this system. Therefore we simplified the ABN by preserving only 2 of the additional possible interactions (Figure 4e, SCL and Fli each activate GATA2) and considered all interactions between X and Y and the four components EKLF, Fli1, cjun and Gfi1 as possible (Supplementary Figure S1).

Even on this reduced model, brute-force simulation failed to identify a single valid model in over 5 days of computation, while RE:IN identified 2 minimal models in ~7 s (Figure 4g). In contrast, caspo identified 264 minimal models in about 5 s. The difference is owing to some of the constraints, which could not be represented directly in caspo. When we modified the ABN so that all considered interactions were marked possible, and relaxed the assumption that each component requires at least one activator to be ‘on’, then RE:IN also identified 264 minimal models. These are similar, but not equivalent, to the set generated using caspo. The difference is possibly due to our restricted regulation conditions compared with the general Boolean update functions considered by caspo (Supplementary Material).

The comparison of a brute-force, simulation-based search, an ASP-based tool and our SMT-based method highlights several important differences between approaches. First, while the brute-force approach can enumerate the entire set of concrete networks for small ABNs, this strategy quickly becomes unfeasible as non-deterministic choices (possible interactions, multiple regulation conditions, unspecified initial states or asynchronous updates) are introduced. In contrast to the ASP approach, which focuses on optimization, our approach focuses predominantly on checking whether consistent models exist. Further, we can use this technique to formulate predictions and test properties of cABNs, with enumeration of concrete models and minimal networks also supported. Thus, the identification of the entire set of minimal networks could be more expensive using RE:IN than caspo. However, our method provides direct strategies for incorporating prior knowledge, such as definite interactions or restrictions on regulation conditions, and supports richer observations, such as cyclic behavior (yeast cell cycle example). When certain constraints not easily incorporated in caspo are relaxed, the two approaches generate similar results, where small differences can be attributed to the richer Boolean update functions considered in caspo.

## Discussion

We present a methodology for the synthesis and analysis of logical models as biological programs, in order to explain and predict cellular decision making. We employ interaction networks as the framework for explaining how computation is performed by a cell, where the critical components are variables of the biological program, which implicitly define the cell state. Interactions indicate the flow of information between components, dynamically constrained by logical regulation conditions. The framework enables us to provide a mechanistic explanation of how a cell translates input signals into a defined output, i.e., a decision. Crucially, we only consider models that fully recapitulate experimental observations, which are thus an integral and explicit part of the program definition that clearly define the biological behavior we seek to explain. As part of this methodology we define a cABN to be the formal representation of a biological program, and capture all mechanisms consistent with available knowledge.

Our method is applicable to the study of a broad range of biological processes, and helps address a variety of biological questions. It enables a modeler or experimentalist starting from the experimental data alone to construct and analyze a cABN by representing the biological knowledge within our framework (Figure 1). By defining a finite set of regulation conditions as an abstraction of detailed regulatory mechanisms, we enable interactions and dynamics to be treated separately. This, together with the intuitive language for encoding cABNs (Figure 2), makes the approach simple to apply, and makes all assumptions explicit. The overall methodology is implemented in the freely available tool RE:IN, with the required computational power in the cloud. Through the case studies, we illustrate how to identify and verify a biological program against observed behaviors (e.g., expression patterns, time course data, steady states and cycles), to expose interaction redundancy, or to search for novel interactions or input signals when the observed behavior cannot be explained. Indeed, revisiting these studies using our approach reveals novel insights that are in agreement with recent evidence in the literature.

Among several modeling approaches for biological networks,^2^ we focus on Boolean models, which provide sufficient expressive power to capture important system properties, while allowing scalable analysis. The Boolean formalism has already proved useful for the study of various systems,^16^ and offers an attractive starting point as the most parsimonious (Occam’s Razor) explanation of complex system behavior. To a degree, it also abstracts away from experimental noise, for example when sufficient expression is observed regardless of the precise measurement. However, our approach requires all qualitative observations to be reproduced exactly, and noise of sufficient magnitude (causing a component to be observed in the incorrect state) could impact our results. Similar robustness issues have been considered as part of other approaches.^33,34^ On the other hand, noise that is inherent to a biological mechanism could be incorporated and studied in our framework as non-determinism, using asynchronous updates or by introducing additional components with unspecified initial states. When a Boolean discretization is too coarse, a multilevel description of component states could be considered,^1,35,36^ and such extensions are compatible with our SMT-based approach.

Our approach incorporates automated network construction and analysis within the same reasoning framework, whereas alternative reconstruction or training approaches^34,37–39^ often require separate analysis tools. Simulation provides one such analysis strategy.^17,40–43^ However, as only concrete models can be simulated, the ABNs we consider would have to be exhaustively sampled to instantiate possible interactions, regulation conditions and initial states, which becomes impractical due to the combinatorial explosion of concrete models as increasingly complex ABNs are considered.

Deciding that a concrete model is consistent with experimental observations is also challenging, especially for asynchronous models. Formal methods or state space analysis (as in ref. 12) provide a strategy for dealing with this, by reasoning about all executions of the system from a set of initial states to verify model behaviors, thereby eliminating the need for simulation. For example, model checking methods are implemented in tools such as SMBioNet^36^ and BIOCHAM^43,44^ for the analysis of biological networks. Despite this, expensive enumeration would still be required to deal with abstract models.

In contrast, our approach permits the exhaustive characterization of the complete set of consistent networks from an ABN, by encoding and solving satisfiability problems. This enables the integration of partial knowledge of network topology and regulation mechanisms, as well as experimental data where only a subset of the components are observed. The scalability of SMT solvers allows us to analyze models of a realistic size, and together with the generality of the SMT problem enables potential future extensions of our method for multilevel logical models, extended regulation condition definitions or analysis questions. Alternative technologies used to address similar constraint problems include Answer Set Programming (ASP)^45^ and Constraint Logic Programming (CLP).^46,47^

Another feature of our method is that we formulate predictions only when all consistent networks are in agreement, which limits the bias of using only one arbitrary concrete model. As a result, generally, fewer predictions are generated, but it is already understood that an ensemble of models provides more robust predictions than a single representation.^48^ Where different models within the set support the hypothesis and the null hypothesis (preventing a prediction from being made) a potential experiment could be performed to eliminate one of these subsets, and refine the cABN further. Identifying such discriminating experiments automatically is a potential future extension to RE:IN and this methodology. Furthermore, we have begun to investigate biological programs that reconfigure throughout development, which requires us to adapt RE:IN for the synthesis of switching networks.^49^

In light of the fact that the use of formal methods to further understanding of biological systems is gaining momentum,^7,9,33,47,50,51^ we anticipate that automated reasoning, and in particular the approach we present here, could be integrated into the process of combining knowledge, formulating hypotheses, and designing new experiments to elucidate further the nature of biological programs. In our analysis, the entire set of consistent models, rather than a single mechanism, describes the biological program. We speculate that this may not simply be an artefact of our approach but rather relate to how a biological program is realized in individual cells. Given that there are multiple genes and interactions with redundant function, different cells could run consistent variants of the program to obtain equivalent behavior. This could explain the inherent heterogeneity and robustness of biological processes.

## Materials and methods

### Formal definitions

Our methodology deals with a class of logical models where each component exists in one of two possible states: active or inactive. Such models can be seen as Boolean abstractions of more detailed descriptions, where the component states vary over multiple discrete values, or are represented by a continuous quantity. In the following, we use B={⊤,⊥} to denote the Boolean values ⊤ (true) and ⊥ (false), which we also represent by 1 and 0, respectively. Given a set *S* we use |*S*| to denote the cardinality (the number of elements) of *S*.

#### Definition 1 (Boolean Network)

*A Boolean network is a tuple* B=(C,F) of a set of components *C* and a set of update functions $F=\left\{f_{c}:B^{\left|C\right|}\to B|c\in C\right\}$, where *f_c_* is the update function for component *c∈C*.

#### Definition 2 (Transition System)

*A transition system is a tuple* T=(Q,T), where Q is the set of states and T:Q×Q→B is a transition relation.

Given states *q*, *q*′∈*Q*, a transition from *q* to *q*′ is allowed if and only if *T*(*q*, *q*′) holds and, therefore, the relation *T* describes the transitions that are valid in the system. We consider deadlock free systems, i.e., where ∀*q*∃*q*′. *T*(*q*, *q*′). A transition system is deterministic if and only if at most a single valid transition exists for each state, and otherwise it is nondeterministic. A sequence of states *q*_0_, *q*_1_, …, *q*_*K*_ represents an execution (a trajectory) of T if and only if a transition from each state to the subsequent one is possible (i.e., ∧_*i*=0…*K*−1_*T*(*q*_*i*_, *q*_*i*+1_)).

Given a Boolean network B=(C,F), let $T_{B}=\left(Q_{B},T_{B}\right)$ denote the transition system we use to describe the dynamics of **B**. The set of states of $T_{B}$ is defined as $Q_{B}=B^{\left|C\right|}$ and captures all possible unique configurations of the components from *C*. Given a system state $q\in Q_{B}$ and a component *c*∈*C*, we use q(c)∈B to denote the state of *c*. Each Boolean update function *f*_*c*_ defines how the state of component *c* is updated, given the current states of all components. For example, given components *c*, *c*′, *c*″, the update function *f*_*c*_(*q*)=*q*(*c*′)∧*q*(*c*″) describes a rule where component *c* will be active in the next state if and only if both components *c*′ and *c*″ are active in the current state. A choice between two different assumptions is commonly applied to define the dynamics of Boolean networks in terms of the application of the update functions for each component. Under synchronous updates, it is assumed that the state of each network component is updated at each step. The transition relation for a synchronous Boolean network is defined as

(1)∀q,q′∈Q.T(q,q′)↔∧c∈Cq′(c)=fc(q),

where *q* and *q*′ represent the current and next system state. In contrast, under asynchronous updates, it is assumed that only the state of a single network component is updated per step, while all other components remain unchanged, and the choice of which component to update is nondeterministic. This leads to the following transition relation definition for asynchronous Boolean networks:

(2)∀q,q′∈Q.T(q,q′)↔∨c∈C(q′(c)=fc(q)∧∧c′∈C,c′≠cq′(c′)=q(c')).

Thus, transition system $T_{B}$ is deterministic if **B** is a synchronous Boolean network, assuming deterministic update functions. In general, $T_{B}$ is nondeterministic if **B** is asynchronous.

An example of a Boolean network **B** together with the transition system $T_{B}$ is presented in Supplementary Figure S2, where we visualize both these systems as graphs. The graph representing **B** captures information about the components, interactions and update functions in the system. In contrast, the graphs representing $T_{B}$ indicate the different states (unique configurations) that the system can exist in, together with the possible transitions that give rise to its dynamical behavior. In practice, the construction of such explicit transition system representations is often not feasible due to the large number of states. For example, a Boolean network with |*C*|=15 components contains 2^|*C*|^=32,678 states and this number grows exponentially as additional components are included.

### Network topology

Let *C* denote the finite set of critical components. Then $Q=B^{\left|C\right|}$ is the set of states of the system and q(c)∈B denotes the state of component *c*∈*C* in state *q*∈*Q*. Let I:C×C×B→B denote the set of directed, **definite** interactions between the components *C*, labeled with a regulation sign (⊤ for positive and ⊥ for negative). Similarly, let $I^{?}:C\times C\times B\to B$ denote the set of directed, **possible** interactions and assume that an interaction can be either definite or possible but not both (i.e., $I\cap I^{?}=\emptyset$). For example, given components *c*, *c*′∈*C*, (*c*, *c*′, ⊤)∈*I* denotes the presence of a definite, positive interaction from *c* to *c*′, while (*c*, *c*′, ⊥)∈*I*^?^ denotes the possibility of a negative interaction from *c* to *c*′.

The set of components *C*, together with the definite and possible interactions *I* and *I*^?^ between elements from *C* defines the abstract network topology we consider (Figure 1, panel 3). This representation describes $2^{\left|I^{?}\right|}$ unique *concrete network* topologies, in which all interactions are marked as definite.

To examine concrete networks, we introduce the functions pos :C×C→B and neg :C×C→B. Given components *c*, *c*′∈*C*, *pos*(*c*, *c*′) indicates that a positive interaction between *c* and *c*′ was selected for the particular concrete network. Similarly, *neg*(*c*, *c*′) indicates that a negative interaction was selected. Clearly, (*c*, *c*′, ⊤)∈*I*→*pos*(*c*, *c*′) and (*c*, *c*′, ⊥)∈*I*→*neg*(*c*, *c*′), i.e., definite interactions are always selected. For all other possible interactions, the functions *pos* and *neg* represent choice variables.

### Regulation conditions

The network topology alone does not capture information about system behavior over time. Rather, we must construct a dynamical model by representing explicitly the rules that govern how the system transitions between different states during executions. Arbitrary Boolean update rules might be ‘too rich’ and describe behaviors not observed in biological systems.^18^ Therefore, in the following, we consider a subset of templates called regulation conditions, which describe qualitatively different regulation mechanisms. Each regulation condition must be applicable to network topologies that contain both definite and possible interactions. This is not straightforward if named regulators are used in the update functions, as a given regulatory interaction might not be included in some of the concrete models. Therefore, we define these rules only in terms of whether none, some, or all activators (repressors) of a given target are present in a given state, assigning equal significance to each regulator. We thereby guarantee that the dynamical rules we consider are consistent with the abstract network topology.

We consider all regulation conditions consistent with the following assumptions.

1. A target is activated when all of its activators are present (i.e., active) and none of its repressors are present (i.e., inactive). Similarly, a target is deactivated when all repressors are present and no activators are present.

This guarantees that the state of the target depends on the state of its regulators and is not permanently active or inactive. We introduce a similar assumption for targets that have only activators (they are not repressible) and only repressors (they are not inducible).

2. We assume that each regulation condition is monotonic. For example, if a target requires only one activator to be activated, then any greater number of activators will activate that target, assuming no change in the state of the repressors.

We formally define the set of 18 regulation conditions consistent with these assumptions in the following. Given as network topology (*I*, *I*^?^, *C*), component *c*∈*C* and a state *q*∈*Q*, we define the terms

$$\begin{array}{ccc} NotInducible\left(c\right) & ≜ & ∄c\prime \in C.\;pos\left(c\prime ,c\right)\\ & & \left(\mathrm{component}\;c\;\mathrm{is}\;\mathrm{not}\;\mathrm{inducible}\right)\\ NotRepressible\left(c\right) & ≜ & ∄c\prime \in C.\;neg\left(c\prime ,c\right)\\ & & \left(\mathrm{component}\;c\;\mathrm{is}\;\mathrm{not}\;\mathrm{repressible}\right)\\ AllActivators\left(c,q\right) & ≜ & \neg NotInducible\left(c\right)\wedge \wedge_{c\prime \in C}\;pos\left(c\prime ,c\right)\to q\left(c\prime \right)\\ & & \left(\mathrm{all}\;\mathrm{activators}\;\mathrm{of}\;c\;\mathrm{are}\;\mathrm{present}\;\mathrm{in}\;q\right)\\ NoActivators\left(c,q\right) & ≜ & \wedge_{c\prime \in C}\;pos\left(c\prime ,c\right)\to \neg q\left(c\prime \right)\\ & & \left(\mathrm{no}\;\mathrm{activators}\;\mathrm{of}\;c\;\mathrm{are}\;\mathrm{present}\;\mathrm{in}\;q\right)\\ AllRepressors\left(c,q\right) & ≜ & \neg NotRepressible\left(c\right)\wedge \wedge_{c\prime \in C}\;neg\left(c\prime ,c\right)q\left(c\prime \right)\\ & & \left(\mathrm{all}\;\mathrm{repressors}\;\mathrm{of}\;c\;\mathrm{are}\;\mathrm{present}\;\mathrm{in}\;q\right)\\ NoRepressors\left(c,q\right) & ≜ & \wedge_{c\prime \in C}\;neg\left(c\prime ,c\right)\to \neg q\left(c\prime \right)\\ & & \left(\mathrm{no}\;\mathrm{repressors}\;\mathrm{of}\;c\;\mathrm{are}\;\mathrm{present}\;\mathrm{in}\;q\right) \end{array}$$

Note that by negating the functions defined above, we obtain additional expressions. For example, ¬*AllActivators*(*c*, *q*) indicates that no activators, or some but not all activators, of *c* are present in state *q*. Using these expressions, we define the following regulation condition templates:

$$\;\begin{array}{ccc} R_{0}^{\prime }\left(c,q\right) & ≜ & AllActivators\left(c,q\right)\wedge \;NoRepressors\left(c,q\right)\\ R_{1}^{\prime }\left(c,q\right) & ≜ & \neg NoActivators\left(c,q\right)\wedge \;NoRepressors\left(c,q\right)\\ R_{2}^{\prime }\left(c,q\right) & ≜ & AllActivators\left(c,q\right)\wedge \neg AllRepressors\left(c,q\right)\\ R_{3}^{\prime }\left(c,q\right) & ≜ & \left(NoRepressors\left(c,q\right)\wedge \neg NoActivators\left(c,q\right)\right)\vee \\ & & \left(\neg AllRepressors\left(c,q\right)\wedge \;AllActivators\left(c,q\right)\right) \end{array}$$

$$\begin{array}{ccc} R_{4}^{\prime }\left(c,q\right) & \;≜ & AllActivators\left(c,q\right)\\ R_{5}^{\prime }\left(c,q\right) & \;≜ & AllActivators\left(c,q\right)\vee (NoRepressors\left(c,q\right)\wedge \neg NoActivators\left(c,q\right))\\ R_{6}^{\prime }\left(c,q\right) & \;≜ & \neg NoActivators\left(c,q\right)\wedge \neg AllRepressors\left(c,q\right)\\ R_{7}^{\prime }\left(c,q\right) & \;≜ & \left(\neg NoActivators\left(c,q\right)\wedge \neg AllRepressors\left(c,q\right)\right)\vee AllActivators\left(c,q\right)\\ R_{8}^{\prime }\left(c,q\right) & ≜ & \neg NoActivators\left(c,q\right) \end{array}$$

$$\begin{array}{ccc} R_{9}^{\prime }\left(c,q\right) & ≜ & NoRepressors\left(c,q\right)\\ R_{10}^{\prime }\left(c,q\right) & ≜ & NoRepressors\left(c,q\right)\vee \left(\neg AllRepressors\left(c,q\right)\wedge \;AllActivators\left(c,q\right)\right)\\ R_{11}^{\prime }\left(c,q\right) & ≜ & NoRepressors\left(c,q\right)\vee \left(\neg NoActivators\left(c,q\right)\wedge \neg AllRepressors\left(c,q\right)\right)\\ R_{12}^{\prime }\left(c,q\right) & ≜ & \neg AllRepressors\left(c,q\right)\\ R_{13}^{\prime }\left(c,q\right) & ≜ & NoRepressors\left(c,q\right)\vee AllActivators\left(c,q\right) \end{array}$$

$$\;\begin{array}{ccc} R_{14}^{\prime }\left(c,q\right) & ≜ & \left(NoRepressors\left(c,q\right)\vee AllActivators\left(c,q\right)\right)\vee \\ & & \left(\neg AllRepressors\left(c,q\right)\wedge \neg NoActivators\left(c,q\right)\right)\\ R_{15}^{\prime }\left(c,q\right) & ≜ & \neg AllRepressors\left(c,q\right)\vee AllActivators\left(c,q\right)\\ R_{16}^{\prime }\left(c,q\right) & ≜ & NoRepressors\left(c,q\right)\vee \neg NoActivators\left(c,q\right)\\ R_{17}^{\prime }\left(c,q\right) & ≜ & \neg AllRepressors\left(c,q\right)\vee \neg NoActivators\left(c,q\right) \end{array}$$

Given these definitions, a component *c* that is non-inducible would satisfy *AllActiv*a*tors*(*c*, *q*), as well as *NoActivators*(*c*, *q*) for any state *q*∈*Q*, and the same holds for non-repressible components. To ensure that non-inducible and non-repressible components are not constantly activated or repressed regardless of the state of their activators, we introduce the following:

$$\begin{array}{ccc} InducibleRegulation\left(c,q\right) & ≜ & [\neg NotInducible\left(c\right)\\ & & \wedge \;NotRepressible\left(c\right)]\to \neg NoActivators\left(c,q\right),\\ RepressibleRegulation\left(c,q\right) & ≜ & \neg NotRepressible\left(c\right)\\ & & \wedge \;NotInducible\left(c\right)\;\wedge \;NoRepressors\left(c,q\right). \end{array}$$

This leads to the the final regulation condition definition:

$$R_{i}\left(c,q\right)\;≜\;\left[R_{i}^{\prime }\left(c,q\right)\wedge \;InducibleRegulation\left(c,q\right)\right]\vee RepressibleRegulation\left(c,q\right)\;\mathrm{for}\;i=0\ldots 17,$$

which defines the set of all 18 regulation conditions consistent with our assumptions. These 18 regulation conditions are represented visually in Supplementary Figure S3.

Two additional rules that are consistent with the requirements of monotonicity and the fact that no named regulators are used are the instant and delayed ‘threshold rule’,^11^ in which the balance of activators and repressors determines whether the component is activated or repressed. The delayed threshold rule specifies that if a target node is active at time *t*, and the total input to the target is zero, then it will be degraded at time *t*=*t*+*t*_*d*_. Here we consider a simplified version of the delayed threshold rule applicable for modeling a self-degradation for components that have no negative regulators when *t*_*d*_=1. Formally, we define these rules as

$$\begin{array}{cc} R_{18}\left(c,q\right)≜(\#A\left(c,q\right)> & \#R\left(c,q\right))\vee (\#A\left(c,q\right)=\#R\left(c,q\right)\\ & \wedge \;q\left(c\right))\;\left(\mathrm{instant}\;\mathrm{threshold}\;\mathrm{rule}\right),\\ R_{19}\left(c,q\right)≜A\left(c,q\right)> & \;\#R\left(c,q\right),\;\left(\mathrm{delayed}\;\mathrm{threshold}\;\mathrm{rule}\right) \end{array}$$

where #*A*(*c*, *q*) (#*R*(*c*, *q*)) denotes the number of activators (repressors) of component *c* that are active at state *q*.

The set of 18 regulation conditions we define, together with the two threshold rules, completes the set of 20 regulation conditions.

### Abstract Boolean network

#### Definition 3 (Abstract Boolean Network)

*An abstract Boolean network (ABN) is a tuple* $A=\left(C,I,I^{?},r\right)$, where

*● C* *is the finite set of components*,

*●* I:C×C×B→B *is the set of definite (positive and negative) interactions between the components from C*,

*●*$I^{?}:C\times C\times B\to B$ *is the set of possible (positive and negative) interactions, and*

*●* $r=\left\{r_{c}|c\in C\right\}$ *where r_c_⊆R assigns a subset of the regulation conditions (denoted here by R={R_0_ … R_19_}) to each component from C*.

The set of components *C* together with the definite and possible interactions *I* and *I*^?^ define the abstract network topology. A choice from our set of 20 regulation conditions is allowed for each component. When the precise regulation mechanism for a given component is unknown, all regulation conditions can be assigned as possible rules. If prior experimental evidence can eliminate certain regulation mechanisms, then a subset can be assigned. For example, regulation conditions *R*_0_ to *R*_8_ require that an activator is present in order for a component to be activated in the next state. Combined with a requirement on the topology that each component must include one activator (to prevent the case of non-inducible components), this restriction (*r*(*c*)={*R*_0_ … *R*_8_} for some *c*∈*C*) can be used to model the specific regulation mechanisms considered to hold in higher order organisms.

We define an ABN to describe the uncertainty in the precise network topology and regulation rules for each component. An ABN is transformed into a concrete model by selecting a subset of the possible interactions to be included (while all other optional interactions are discarded) and assigning a specific regulation condition for each gene.

Formally, let ${\hat{I}}^{?}\subseteq I^{?}$ denote the set of selected possible interactions, $\hat{I}=I\cup {\hat{I}}^{?}$ denote the set of all selected interactions, which thus includes all definite interactions. Note that, $\left(c,c',⊤\right)\in \hat{I}↔pos\left(c,c\prime \right)$ and $\left(c,c\prime ,⊥\right)\in \hat{I}↔neg\left(c,c\prime \right)$—that is, the selected interactions from $\hat{I}$ determine the functions *pos* and *neg* used for the definition of all regulation conditions. Let ${\hat{r}}_{c}\in r_{c}$ denote the specific (single) regulation condition that was selected for each component *c*∈*C* and $\hat{r}=\left\{{\hat{r}}_{c}\left|c\in C\right|\right\}$ denote all the selected regulation conditions.

We define the semantics of such a concrete model in terms of a transition system $T_{A}=\left(Q,T\right)$, where $Q=B^{\left|C\right|}$ is the set of states (q(c)∈B is the state of component *c* in state *q*∈*Q*). As for Boolean networks, we consider both synchronous and asynchronous semantics. For synchronous systems, the transition relation T:Q×Q→B is defined as

(3)∀q,q′∈Q.T(q,q′)↔∧c∈Cq′(c)=rˆc(c,q).

For asynchronous systems, the transition relation is defined as

(4)∀q,q′∈Q.T(q,q′)↔∨c∈C(q′(c)=rˆc(c,q)∧∧c′∈C,c′≠cq′(c′)=q(c′)).

The semantics of an ABN can be understood in terms of the (non-deterministic) choice of possible interactions ${\hat{I}}^{?}$ and the choice of a regulation conditions $\hat{r}$, together with the transition system T representing the resulting concrete model. Thus, an ABN captures the trajectories of all the concrete models it can be transformed into and can be seen as representation of the finite set of $n=2^{\left|I^{?}\right|}\cdot \prod_{c\in C}\left|r_{c}\right|$ concrete models $A=\left\{B_{0}\ldots B_{n-1}\right\}$, corresponding to different choices of ${\hat{I}}^{?}$ and $\hat{r}$. Here we denote each concrete model as $B_{i}$, since these models can be represented as a Boolean network, where the choice of update functions is restricted to our 20 regulation conditions. Each concrete model is represented by the transition system $T_{B_{i}}$ and the ABN is represented by transition system $T_{A}=T_{B_{i}}$ where the choice of *i* is nondeterministic.

#### Remark 1

Input signals to an ABN will not have any defined regulators and will be constantly active or inactive depending on the choice of regulation conditions (see Supplementary Figures S3a and S4a,b). This property can be used to model self-degrading (self-activating) signals, which are active (inactive) only during the initial state of an experiment (in the case of the yeast cell cycle model, we used the delayed threshold rule for this purpose). To ensure that an input signal is sustained throughout each experiment (either as active or inactive depending on the initial value) we include a single definite self-activation (Supplementary Figure S4c). Alternatively, an oscillating signal can be modeled by including a single definite self-repression interaction (Supplementary Figure S4d).

### Experimental observations

Some of the unique concrete models represented by an ABN might produce behavior that is consistent with experimental observations of the modeled biological system. However, other concrete models might not be consistent, and thus we seek to impose constraints that eliminate these mechanisms. In the following, we present our approach for defining and incorporating constraints over the behavior (the possible trajectories) of an ABN.

We consider reachability properties over the states of various components at different steps during executions of the system. We seek to formalize observations obtained from different experiments, denoted by the set *E*, where each experiment *e*∈*E* represents a different execution of the system. We construct observations using terms (*e*, *n*, *c*, *v*), where

*● e*∈*E* is the experiment label,

*● n*∈0 … *K* denotes a specific time step,

*● c*∈*C* denotes a component of the ABN, and

*●* v∈B represents the observed state of component *c*.

Let *t*=*q*_0_, …, *q*_*K*_ denote a trajectory of the transition system T for one of the concrete models represented by an ABN, A. Trajectory *t* satisfies the term (*e*, *n*, *c*, *v*) if and only if *q*_*n*_(*c*)=*v* and we require that, for all experiments *e*∈*E*, there exists a trajectory *t*_*e*_ that satisfies all the terms labeled by *e*.

As an example, consider an experiment where component, *c*, was initially observed to be inactive but then observed to be active at a later time point (after 20 steps). Both of these observations describe the same execution of the system and are therefore denoted by the same experiment label, *e*. To represent this, we construct the expression (*e*, 0, *c*, ⊥)∧(*e*, 20, *c*, ⊤) requiring the existence of a trajectory that recapitulates these observations. This trajectory corresponds to an explanation of how the system is capable of reproducing the observed behavior. Multiple labels allow us to represent different experiments, for example when the system is initialized in different states. Note that the expressions related to different labels, for example *e* and *e*′, are not necessarily mutually exclusive and, therefore, the same trajectory might satisfy both.

In addition, we use the terms *KO*(*e*, *c*, *v*) and *FE*(*e*, *c*, *v*) to define knockout and forced expression perturbations, which are assigned to a given experiment and component but do not depend on time. These perturbations modify the dynamics of the system along trajectory *t*_*e*_, where component *c* is always active (forced expression) or inactive (knockout) when *v*=⊤, regardless of the regulation conditions for *c* or the state of each of its regulators (the update rules are applied as before when *v*=⊥).

Finally, we introduce the constraint *Fixpoint*(*e*, *n*) to indicate that the trajectory *t*_*e*_, satisfying all constraints labeled by *e*, must reach a fixed point at step *n*. In other words, the only possible transition from the state *q*_*n*_ of *t*_*e*_ (reached at time step *n*) is a self-loop.

Different terms (*e*, *n*, *c*, *v*), *KO*(*e*, *c*, *v*), *FE*(*e*, *c*, *v*) and *Fixpoint*(*e*, *n*) are combined into logical expressions using the operators {∧, ∨, ⇒, ⇔, ¬}, which allows us to formalize various experimental observations.

### Constrained abstract Boolean network

The ABN can be represented by transition system $T_{A}=T_{B_{i}}$ where the choice of *i* is nondeterministic. However, not all choices of *i* correspond to concrete models that can reproduce the experimentally observed behavior. Therefore, we define the concept of a constrained abstract Boolean network (cABN) as a representation of the set of concrete models that are consistent with experimental observations.

#### Definition 4 (Constrained Abstract Boolean Network)

*A constrained abstract Boolean network (cABN) is a tuple* $A^{c}=\left(C,I,I^{?},r,E,K,F,O\right)$*, where*

●*the components C, definite and possible interactions I and I*^*?*^ *and regulation condition assignment r define an ABN,*

*●E is a set of experiment labels*

*●*K:E×C→B *defines whether there is a knockout perturbation of component c∈C in experiment e∈E,*

*●*F:E×C→B *defines whether there is a forced expression perturbation of component c∈C in experiment e∈E, and*

*●O is an expression representing experimental observations constructed using the terms defined in the previous section.*

As for ABNs, a concrete model of a cABN is defined by selecting a subset of the possible interactions ${\hat{I}}^{?}$ and assigning a specific regulation condition ${\hat{r}}_{c}$ for each component *c*. In addition, the perturbations $\hat{K}$ and $\hat{F}$ must be defined for each experiment *e*∈*E* and component *c*∈*C*. Initially, these perturbations might be unknown or only partially constrained through the experimental observations *O* using the terms *KO*(*e*, *c*, *v*) and *FE*(*e*, *c*, *v*). The dynamics of such a concrete model are defined through a transition system T=(Q,T), where the set of states is the same as for an ABN (i.e., $Q=B^{\left|C\right|}$) but the transition relation T:Q×Q×E→B differs for each experiment *e*∈*E* depending on the perturbations *K* and *F*. For synchronous systems, we define *T* as

(5)∀q,q′∈Q,e∈E.T(q,q′,e)↔∧c∈Cq′(c)=[rˆc(c,q)∧¬K(e,c)]∨F(e,c),

which is related to the definition in Equation 3. The definition for the transition relation of asynchronous system is defined similarly by extending Equation 4 to capture the perturbations *K* and *F*. Note that *K* and *F* could also be introduced for ABNs and BNs to model perturbations in these systems.

A cABN can be viewed as a representation of the finite set of concrete models $A^{c}=\left\{B_{0}\ldots B_{m}\right\}$ with the constraint that, for any valid concrete model $B_{i}$, the transition system $T_{B_{i}}$ representing $B_{i}$ satisfies *O* (denoted as $T_{B_{i}}=O$). In other words, for all experiments *e*∈*E*, there exists a trajectory *t*_*e*_ of $T_{B_{i}}$ that satisfies all the constraints for experiment *e* from *O*. Thus, a cABN $A^{c}=\left(C,I,I^{?},r,E,K,F,O\right)$ corresponds to a subset of the models represented by the ABN $A^{c}=\left(C,I,I^{?},r\right)$ that is consistent with the experimental observations from *O*, i.e., $A^{c}=\left\{B\in A\left|B⊨O\right|\right\}$. Note that, in general, a cABN or an ABN represent a large number of concrete models that cannot be enumerated efficiently or represented explicitly. Therefore, in the following section we propose a symbolic representation of ABNs and cABNs.

### SMT encoding

First, we focus on the following problem, which is central to the analysis questions supported by our methodology:

#### Problem 1 (cABN synthesis)

*Given a cABN* $A^{c}=\left(C,I,I^{?},r,E,K,F,O\right)$*, find one concrete model that is consistent with all experimental observations.*

The solution to Problem 1 amounts to finding ${\hat{I}}^{?}$ and $\hat{r}$, a subset of possible interactions and a specific regulation condition for each component, such that there exists a trajectory *t*_*e*_ of the transition system representing the resulting concrete model that satisfies the experimental observations associated with each label *e*. We encode this as a Satisfiability Modulo Theories (SMT) problem and apply the SMT solver Z3^22^ to obtain the solution.

An SMT problem amounts to deciding whether a logical formula is satisfiable (i.e., there exists a valuation of all variables, for which the formula evaluates to true). SMT extends the classical Boolean satisfiability problem (SAT), which deals only with Boolean formulas, and allows the use of additional theories such as bit vectors. To apply the SMT solver Z3 and the bit vector decision procedures it implements,^21^ we encode Problem 1 as a logical expression using bit vectors.

Given $A^{c}=\left(C,I,I^{?},r,E,K,F,O\right)$, we first encode the set of possible interactions *I*^?^ as the bit vector $interactions\in B^{\left|I^{?}\right|}$. Each position in *interactions* encodes the Boolean choice variable representing whether the particular interaction is selected or not. Similarly, we encode the regulation condition for each component *c*∈*C* as the bit vector $regulation_{c}\in B^{5}$ with the constraint

(6)∨i∈ids(rc)regulationc=i,

where *ids*(*r*_*c*_) represents the indexes of the regulation conditions allowed for component *c*. A valuation of *interactions* and each *regulation*_*c*_ represents one concrete model from $A^{c}$, where ${\hat{I}}^{?}$ is determined by *interactions* and ${\hat{r}}_{c}$ is determined by *regulation*_*c*_ for each *c*∈*C*. However, at this point *interactions* and *regulation*_*c*_ are symbolic representations without a concrete valuation.

For each experiment *e*∈*E* we can encode the perturbations *K*(*e*, ·) and *F*(*e*, ·) as bit vectors of size |*C*|. This would allow perturbations for each component as part of each experiment to be specified using the observations *O*, but the choice of perturbation remains unconstrained (non-deterministic) unless such observations are provided. However, perturbations are often considered only for a small number of components, which requires many additional, trivial observations (e.g., *KO*(*e*_0_, *c*, ⊥)∧*KO*(*e*_1_, *c*, ⊥)∧ … when no knockout perturbations are considered for component *c* as part of any experiment). To simplify our encoding, we designate that only a subset of the components to be considered for knockout (*C*_*K*_) or forced expression (*C*_*F*_) perturbations and define the *K*(*e*, ·) and *F*(*e*, ·) bit vectors to be of sizes |*C*_*K*_| and |*C*_*F*_|, respectively. It should be noted that once a component is included in the subsets *C*_*K*_ and/or *C*_*F*_, a knockout or forced expression can be assigned by the solver in such a way that all observations from *O* are satisfied. Thus, additional observations must be introduced for such components to indicate the lack of perturbation in certain experiments.

The set of states of an ABN (or cABN) is $Q=B^{\left|C\right|}$. Therefore, a given state can be represented conveniently as a bit vector of size |*C*|. Given such a representation for two states *q*, *q*′∈*Q* and experiment *e*∈*E*, the bit vectors *interactions* and *regul*a*tion*_*c*_ allow us to encode the transition relation for synchronous systems defined in Equation 5 (the same approach applies for asynchronous systems). To encode trajectories, we follow an approach inspired by Bounded Model Checking,^52^ where we consider a finite number of steps and ‘unroll’ the transition relation *T*. Given an experiment *e*∈*E*, we encode each state *q*_*i*_ of the trajectory *t*_*e*_=*q*_0_ … *q*_*K*_ as a bit vector and assert the constraint

(7)∧i=0…K−1T(qi,qi+1,e).

Once we have an encoding of trajectory *t*_*e*_ for each experiment *e*∈*E*, we assert the constraints from *O* over the states of these trajectories. For terms (*e*, *n*, *c*, *v*) this amounts to a constraint *q*_*n*_(*c*)=*v* for trajectory *t*_*e*_. Terms *KO*(*e*, *c*, *v*) and *FE*(*e*, *c*, *v*) correspond to constraints over the bit vectors representing the perturbations of each experiment.

Finally, we describe the encoding of fixed point constraints *Fixpoint*(*e*, *n*). For synchronous systems, this corresponds to a constraint that state *q*_*n*_ of trajectory *t*_*e*_ (corresponding to experiment *e*∈*E*) includes a self-transition, i.e., *T*(*q*_*n*_, *q*_*n*_, *e*). As the transition system is deterministic, requiring such a self-transition guarantees that this is the only outgoing transition from *q*_*n*_ and therefore the state is stable. For asynchronous systems, which can generate non-deterministic behavior, in general it is possible that multiple outgoing transitions exist at a state. In this case, we assert a constraint for a self-transition using the transition relation for an equivalent synchronous system, instead of using the asynchronous transition relation, which is necessary and sufficient to guarantee that state *q*_*n*_ is stable.

### Analysis procedures

Our methodology supports a number of analysis procedures, described in the following.

#### System diameter

In many cases, we are interested in expressing end-point behavior, where an initial and final configuration of (a subset of) the system components are given. A parameter that must be specified for such experimental observations is the trajectory length that is considered. Specifying a trajectory length that is too short might exclude valid mechanisms from the cABN, which require a larger number of steps to reach the required configuration.

While an appropriate trajectory length is often hard to specify, the recurrence diameter (the longest loop-free trajectory of the system) can be used as an overapproximation that guarantees that no valid mechanisms are excluded. To find the recurrence diameter, we consider a trajectory *t*=*q*_0_, …, *q*_*K*_ with the constraint that *q*_*i*_≠*q*_*j*_ for *i*=0 … *K*−1, *j*=*i*+1 … *K*. We then use the SMT solver to check if such a loop-free trajectory *t* exists and increase *K* until this is no longer the case, at which point *K* represents the recurrence diameter.

By asserting all experimental observations from *O*, this procedure allows us to compute the longest loop-free trajectory for any of the mechanisms described by the cABN. Furthermore, if no perturbation constraints are asserted for *t*, then the longest possible diameter under any possible perturbations is computed. However, additional constraints on perturbations or the initial configurations of components could be asserted for *t*, for example, to consider only initialized trajectories as part of the diameter computation.

In general, the computation of a recurrence diameter is a challenging problem and the procedure we apply requires prohibitively long time for certain systems (e.g., the asynchronous system from the myeloid progenitor case study) but allows us to obtain this parameter for other systems (e.g., a diameter of 28 steps was identified for the budding yeast cell cycle model). Besides providing a convenient strategy for specifying experimental observations such that no valid mechanism are excluded, the computation of a system’s diameter also reveals an important property of the model that is being investigated (i.e., the largest possible number of steps before the system reaches a potential attractor).

#### Concrete model enumeration

Thus far, we have considered the problem of finding a single concrete mechanism from the set of consistent mechanisms described by a cABN (Problem 1), but in many cases we are interested in enumerating a number of unique consistent mechanisms. Depending on the application, mechanisms can be considered unique if only the network topology is different, either the topology or the regulation conditions differ, or an alternative explanation for how the experimental observations are achieved is obtained. Formally, we encode each of these cases as follows.

Suppose we have a cABN $A^{c}=\left(C,I,I^{?},r,E,K,F,O\right)$, where *interactions* is the bit-vector representing possible interactions, *regulation*_*c*_ is the bit-vector representing the regulation condition for each component *c*∈*C*, and trajectory *t*_*e*_ is an explanation of how the experimental observations *e*∈*E* are satisfied. A concrete mechanism corresponds to a valuation of *interactions* and all *regulation*_*c*_ and *t*_*e*_.

Let $\overset{¯}{interactions}$, $\overset{¯}{regulation_{c}}$ and $\overset{¯}{t_{e}}$ denote the representation of one concrete mechanism from the cABN. To identify a different consistent mechanism, we assert one of the following additional constraints.

*●* Only the network topology is different:

$$interactions\neq \overset{¯}{interactions}$$

*●* Either the network topology is different or the regulation condition of at least one component is different:

$$interactions\neq \overset{¯}{interactions}\vee \underset{c\in C}{\vee }regulation_{c}\neq {\overset{¯}{regulation}}_{c}$$

*●* Either the network topology, the regulation condition of at least one component, or the trajectory for at least one experiment are different:

$$interactions\neq \overset{¯}{interactions}\vee \underset{c\in C}{\vee }regulation_{c}\neq {\overset{¯}{regulation}}_{c}\vee \underset{e\in E}{\vee }t_{e}\neq {\overset{¯}{t}}_{e}$$

A number of qualitatively different mechanisms can be enumerated by obtaining a solution using the SMT solver, asserting the uniqueness constraints defined above and applying this procedure for a number of iterations or until no additional solutions are possible. When no additional solutions can be generated (a result that is obtained by applying the SMT solver), we guarantee that all possible consistent mechanisms have been enumerated. However, this may not be feasible in practice for a large number of components and possible interactions, which permit a large possible number of solutions. Thus, in the following sections we propose analysis strategies that do not require the explicit enumeration of all consistent mechanisms.

### Minimal models

In certain studies, we are interested in identifying minimal models—the most parsimonious mechanisms from a cABN that explain all experimental observations. More specifically, we focus on mechanisms that involve the smallest number of possible interactions to achieve the specified behavior. We identify such minimal models using the following procedure.

Let $\overset{¯}{interactions}$ denote the valuation of the bit-vector representing the possible interactions selected as part of one concrete, consistent mechanism identified. Let #$\overset{¯}{interactions}$ denote the *cardinality* (the number of individual bits set to ⊤), which represents the number of selected possible interactions. To identify mechanisms with the smallest number of interactions, we first use the SMT solver to identify one consistent mechanism and then enforce the additional constraint

$$\#interactions<\#\overset{¯}{interactions},$$

which guarantees that a mechanism with fewer interactions is identified if one exists. We apply this procedure iteratively until no solutions can be generated, at which point the minimal number of possible interactions that must be included is identified. This procedure can also be combined with concrete model enumeration to identify all minimal models that contain the same number of interactions.

### Required and disallowed interactions

Given a cABN, a required interaction is a possible interaction that appears in all consistent mechanisms, i.e., this interaction must be present to reproduce all experimental observations. Similarly, a disallowed interaction is one that does not appear in any of the consistent mechanisms, i.e., including such an interaction makes it impossible to reproduce the experimental observations. One possible approach for identifying required and disallowed interactions would involve the enumeration of all consistent mechanisms, but this strategy is not feasible in practice when the number of mechanisms from the cABN is large. Therefore, we use the following alternative approach.

Let ${\hat{I}}^{?}$ represent the possible interactions selected for one concrete mechanism. An interaction from the set ${\hat{I}}^{?}$ is potentially a required interaction, given it is present in at least one consistent mechanism. Similarly, any interaction from the set $I^{?}/{\hat{I}}^{?}$ is potentially a disallowed one, since it was not selected in at least one consistent mechanism. To identify required interactions, for each interaction $i\in {\hat{I}}^{?}$ we construct a new cABN with a set of definite and possible interactions *I*′ and *I*^?^′ such that i∉I′ and $i\notin I^{?\prime }$ (i.e., interaction *i* is removed completely from the system). Applying the SMT solver to the modified cABN allows us to guarantee that *i* is required when no solutions can be generated. Similarly, we identify disallowed interactions, by constructing a new cABN with interaction sets *I*′ and *I*^?′^ such that *i*∈*I*′ and $i\notin I^{?\prime }$ for each interaction $i\in I^{?}/{\hat{I}}^{?}$ (i.e., these interactions are included as definite in the modified cABN). Not identifying any solutions using the SMT solver guarantees that none of the consistent models include *i* and, therefore, this interaction is disallowed.

### Predictions

More generally, we are interested in formulating predictions about the behavior of the cABN that hold for all consistent mechanisms—to remove the need to select, and therefore bias toward, one concrete model for analysis of the system. We achieve this using the following approach.

First, we formulate an hypothesis of the behavior of the system that will be investigated, which can be expressed as an experimental observation. For example, such an hypothesis might specify that under a given perturbation the system must eventually stabilize in a state where certain components are inactive. If consistent mechanisms exist that satisfy this new constraint, we then instead encode the null hypothesis (the negation of the hypothesis we are testing), and use the SMT solver to check if any of the consistent mechanisms from the cABN can reproduce this alternative behavior. If this is the case, no prediction can be made, since at least one mechanism supports the null hypothesis. However, in cases where no solutions are found on testing the null hypothesis, we guarantee that all consistent mechanisms support the hypothesis, which leads to a prediction. This approach allows us to consider the entire set of consistent mechanisms from the cABN when generating predictions, avoiding both the implicit bias of a single selected mechanism, as well as the need for solution enumeration.

## Figures and Tables

**Table 1:** A summary of the detail of interactions that can be inferred from different experimental data sources (Supplementary Material)

*Technique*	*Components*	*Directionality*	*Sign*	*Direct*
ChIP-Seq	TF to gene	Yes	No	Yes
Genetic or chemical perturbations followed by gene-expression measurement	TF to gene, signal to gene	Yes	Yes	Yes, if performed in presence of Cycloheximide
Single-cell expression analyses	Any pair of genes/proteins	No	Yes	No
Mass-spectrometry	Interacting proteins, post-transcriptional modifications	Yes	No	Yes

Abbreviations: ChIP, chromatin immunoprecipitation; TF, transcription factor.

**Table 2:** Loss of function of specific genes was predicted to arrest the cell cycle at different phases

*Mutant*	*Prediction*	*Experimental Support*	*Reference (SGD ID)*
Cln3	Prevents transition from Start to G1	*Incorrect prediction*: Cln3 knockdown is found to lead to increased G1 duration	S000000038
MBF or SBF	Prevents transition from G1 to S	Either MBF or SBF knockdown leads to increased g1 duration	S000004172
Clb56 or SBF	Prevents transition from S to G2	Clb56 knockdown causes a delay in the progression through S phase	S000006324
Clb12 or SBF	Prevents transition from G2 to M	(1) SBF knockdown leads to delayed G2/M transition	(1) S000000913
		(2) Clb12 knockdown delays progression through M phase	(2) S000006323
Cdc20	Prevents transition from M to G0	Cdc20 knockdown delays progression through M phase	S000003084

Experimental support for these predictions has been found through the *Saccharomyces* Genome Database (www.yeastgenome.org). Only one prediction was found to be incorrect (Cln3 mutant).

**Table 3:** Through literature search, we found evidence to support six out of the eight new potential interactions identified that enable the temporal dynamics of differentiation to be satisfied (Figure 5c,d)

*Interaction*	*Experimental evidence*
Nkx2.5 --> canWnt	Cambier *et al.*^26^ show that Nkx2.5 regulates cardiac growth, activating Wnt through induction of R-Spondin3, a positive regulator of canonical Wnt.
Mesp1 --| canWnt	David *et al.*^27^ show that Mesp1 induces DKK, an inhibitor of canonical Wnt signaling during cardiovascular differentiation (Mesp1 --> Dkk1 --| cantWnt).
GATAs --> canWnt	Afouda *et al.*^28^ show that inhibition of Gata4 and Gata6 results in reduced expression of cardiac markers and Wnt11.
Bmp2 --> canWnt	Papathanasiou *et al.*^29^ show that BMP2 activates canonical Wnt signaling through induction of LRP-5, a Wnt co-receptor. Rosen^30^ reviews results showing that, in bone development, Bmp2 knockout leads to downregulation of several Wnt pathway components and targets.
Foxc1/2 --| Tbx5	Hilton *et al.*^31^ show that there may be an indirect regulation via Pitx2.
Foxc1/2 --> canWnt	Seo and Kume^32^ show that Wnt expression is absent in Foxc1−/−; Foxc2−/− compound mutants.

This suggests that these may be plausible missing connections in the network governing cardiac development.
