# Supplementary material for: A method to identify and analyze biological programs through automated reasoning
Source: NPJ Syst Biol Appl. 2016 Jul 7;2:16010–. doi: 10.1038/npjsba.2016.10 (PMC5034891; doi:10.1038/npjsba.2016.10)
Supplement: Supplementary Information [file npjsba201610-s1.doc]

# Supplementary Material

##

## Inferring Interactions From Experimental Data

A summary of experiments that can be used to identify the sign and directionality of potential interactions is provided in Table 1.

ChIP-Seq data, or promoter analysis, can provide a list of genes potentially regulated by a given transcription factor. The directionality of an interaction can also be gleaned from genetic perturbation studies, where the effect of inactivation or over-activation of a given component can be measured. One limitation to this approach is that several genes affected by the perturbation could be secondary targets. Chemical perturbation is also an option, via the activation or repression of signalling pathways affecting some of the components of the program. In each case, time series data can inform putative regulations, based on correlations in gene expression.

For the analysis of genetic or chemical perturbations it is ideal to deal with a relatively homogeneous cell population, as a given component can play a different role in different cell states. If the system of interest is intrinsically heterogeneous, single-cell measurements are preferable because they allow us to exploit such heterogeneity to extract information (Moignard et al., 2015). For example, in embryonic stem (ES) cells immunostaining has been used to calculate correlations among key factors (Muñoz Descalzo et al., 2013), whereas single cell qPCR followed by Spearman correlation ranking has been used to identify regulatory elements in the haematopoietic stem cell gene network (Moignard et al., 2013). Again, however, single-cell expression analyses do not tell us the directionality of the putative interactions.

Biochemical interactions such as protein-protein interactions, or post-translation modifications such as phosphorylation or ubiquitination can affect the activity of a component. These can be detected on a global level by Mass Spectrometry. The effect and directionality of such interactions is not always obvious, as phosphorylation activates some proteins and promotes degradation of others. Lastly, it is possible to combine data types to infer possible interactions. For example, ChIP-Seq and genetic perturbation studies have previously been taken together to build a directed and signed Boolean network (BN), with single cell perturbation studies used to infer update functions at each node (Xu et al., 2014).

We assign interactions as definite only if they have significant experimental support. For transcriptional regulation, it is generally accepted that measuring gene-expression shortly after a genetic or chemical perturbation allows you to rule-out secondary effects. In the case of an activation or blockade of a signalling pathway, it is also possible to block protein synthesis using Cycloheximide, to exclude secondary effects formally. Finally, ChIP or promoter assays should also be used to support a direct interaction, to label it as definite. In the case of protein-protein interactions, reciprocal Immuno Precipitation and Fluorescence Resonance Energy Transfer (FRET) represent further experimental validations of interactions inferred from high-throughput studies. For post-translation modification, mutagenesis of individual residues and in vitro assays are generally accepted as strong evidence.

## Comparison of RE:IN With Different Approaches

## To illustrate the power of the method presented in this paper further, we compared it against two alternative approaches, which we first describe below.

### A brute force approach

The first approach involved a naïve, exhaustive, brute-force search of concrete models from a given ABN, where all possible interactions and allowed regulation conditions for each component were systematically instantiated. The initial condition for each experiment was extracted from the set of observations of a cABN and a simulation was performed from each of these initial states. All remaining observations, e.g. final states, associated with each experiment were compared against the results from these simulations in order to determine if the correct behavior was reproduced. In this way, each concrete model from the cABN was explored and labelled as valid or not.

For this comparison, we only considered examples where the initial states of all components were specified in all experiments. In general, when the initial states of components are not fully specified, which is permissible as part of the observations we consider using our method, additional instantiations are required to select a concrete initial state for simulation. For example, applying the brute-force approach to a cABN with components A, B and C and an observation specifying that A and B are initially active but the state of C is not known would require trajectories from both initial states (1,1,1) and (1,1,0) to be explored through simulations. Furthermore, we only considered synchronous ABNs, which allowed the behavior of each concrete, deterministic model from a given concrete initial state to be explored using a single simulation. For asynchronous ABNs, the non-deterministic order of component updates could lead to different behaviors and a large number of simulations would be required to determine if the model is valid, especially when the exhaustive exploration of all possible behaviors is required, as in our analysis.

### CASPO

The second, alternative approach we considered involved the previously developed ‘*Cell ASP Optimized’* (*caspo*) tool (Guziolowski et al., 2013), which is based on Answer Set Programming (ASP). *Caspo* was developed to allow users to identify a set of logical models that are optimal with respect to a given dataset. To use this tool, we encoded the interaction topology of a given ABN as prior knowledge network (PKN), which captures information about the signed interactions between components. Using this representation, it was not clear how to specify certain interactions as definite while others as possible using *caspo*, even though such prior knowledge could, in principle, be incorporated as part of an ASP encoding.

The set of observations from a cABN was represented as a *Minimum Information for Data Analysis in Systems Biology* (*MIDAS*) file supported by *caspo*, which contained information about *treatments* (the states of signals) and induced responses of various components, specified as a *data acquisition* entry specifying the time point and a *data value* entry specifying the measured Boolean activity for each experiment. Since only a specific ‘final’ time point is considered by *caspo*, it was not clear how to specify certain behaviors, such as the cycles we considered in the yeast cell cycle study. Therefore, we restricted our comparison to examples where only the initial and final expressions of all components are observed.

Finally, the more general Boolean update functions considered by *caspo* do not map directly to the regulation conditions we define as part of our approach. Therefore, in the following we compare our method and *caspo* only in terms of the interaction network topologies identified by both approaches.

### Comparisons

First, we considered the simple cABN from Fig. 1. If we consider that both the interaction topology and regulation conditions assigned to each component define unique solutions (e.g. two concrete networks with the same topology but different regulation conditions for a given component are considered distinct), the brute-force simulation approach took approximately 2 minutes to search through all possible 3888 concrete models in order to identify the 1080 models consistent with the observations. In contrast, our SMT-based approach required about 15 seconds to enumerate the same 1080 solutions.

A minimal solution involving three interactions (S1 → A, S2 → B, and B → C) was identified in under 1 second using both *caspo* and our approach. Enumerating all possible topologies is possible using *caspo* by specifying a sufficient topology size tolerance that permits the generation of sub-optimal solutions. This resulted in 6 concrete networks, while RE:IN identified the 8 networks shown in Fig. 1 (panel 8). Both methods performed this analysis also in under 1 second, and consistently identified the activation of C by B as required. In addition, the models generated by *caspo* identified the activation of both A and B by S1 and S2 respectively as required, while these interactions were considered as definite using our approach (see Fig. 1). Interestingly, the two additional solutions identified using our approach that involve a feedback loop between components A and B (A activating B and B activating A) were not identified by *caspo*.

Next, we considered the model of deterministic myeloid differentiation in the presence of unknown signals X and Y, as presented in the main text (Fig. 4). This choice of model was motivated by the fact that the other examples we present in the main text were not directly compatible with brute-force or *caspo* approaches. More specifically, the yeast cell cycle model involved observations of a cyclic behavior that could not be easily expressed as part of the data sets considered by *caspo*. The cardiac development model, on the other hand, did not specify the initial state of all components, making simulation challenging as discussed above. Similarly, the asynchronous myeloid progenitor differentiation models (without signals) could not be easily studied using simulation or *caspo* due to the inherent non-determinism.

Analysing the full myeloid signals model presented in the main text using *caspo* led to memory errors, potentially caused by the complexity of this system and therefore, we simplified it as follows. First, we preserved only two of the additional interactions identified as part of our RE:IN analysis (Fig. 4e, SCL →GATA2, Fli1 →GATA2). We then considered all positive and negative interactions between the hypothetical signals X and Y and only four components (EKLF, Fli1, cjun and Gfi1). All these interactions were considered as ‘possible’ leading to the ABN shown in Fig. S1.


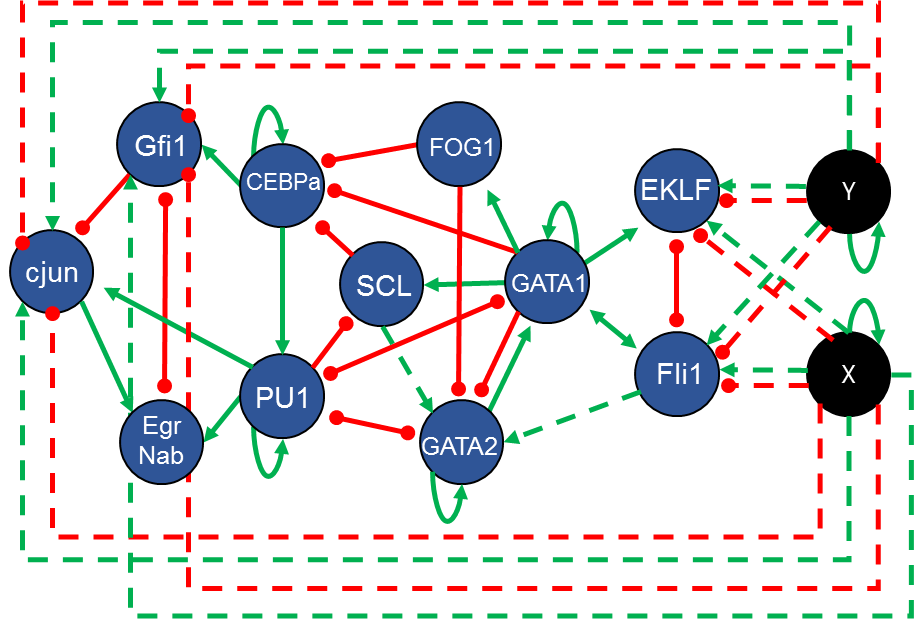


**Figure S1: Reduced deterministic myeolid progenitor differentiation model involving hypothetical signals X and Y.**

Even on this reduced model, the brute-force approach failed to identify even a single valid model in over 5 days of computation. In contrast, RE:IN identified the two minimal networks presented in Fig. 4g in approximately 7 sec. Finally, *caspo* identified 264 minimal solutions in about 5 sec. A larger number of minimal networks were identified by *caspo* because some of the constraints could not be incorporated by the tool. To demonstrate this, we modified the ABN so that all considered interactions were marked as ‘possible’ and the assumption that each component requires at least one activator to be ‘on’ was relaxed. Encoding this modified model in RE:IN allowed us also to identify 264 minimal models, which were similar, but not completely equivalent, to the set generated using *caspo,* as discussed below*.*

Five interactions (Fli1→ GATA1, GATA1 → Fog1, X → Fli1, Y → EKLF and cjun → EgrNab) were included in each of the concrete models identified by both tools and three of these interactions (GATA1 → Fog1, X → Fli1 and Y → EKLF) were confirmed as required using RE:IN. Three additional interactions (EKLF --| Fli1, Fli1 → GATA2 and a self-activation of GATA1), two of which (except the self-activation of GATA1) were confirmed as required, were included in each concrete minimal model identified by RE:IN, but did not appear in any of the solutions identified using *caspo*. This discrepancy could be attributed to the differences between the regulation conditions we consider and the more general Boolean update functions considered by *caspo*. However, two of the three interactions not identified by *caspo* (self-activation of GATA1 and EKLF --| Fli1) were included in the original model from Krumsiek et al. prompting us to consider them as definite in the analysis in the main text, while the third interaction (Fli1 → GATA2) is supported by direct binding evidence from the literature. All other interactions were included in the same number of concrete, minimal models as identified by the two approaches.

The comparison of a brute-force, simulation-based search, the ASP-based *caspo* and our SMT-based method encapsulated in RE:IN highlights several important differences between the approaches. First, while the brute-force approach can enumerate through the entire set of concrete networks for small models, this strategy quickly become unfeasible as additional non-deterministic choices (e.g. possible interactions, multiple regulation conditions allowed for a component, unspecified initial states or asynchronous updates) are introduced in the ABN. The ASP approach of *caspo* focuses on optimization and attempts to find the complete set of minimal networks (in terms of the number of interactions) that best reproduce observed behavior, but a parameter controlling the network size tolerance can be adjusted to generate sub-optimal solutions. In contrast, our approach focuses predominantly on checking whether consistent models exist, and uses this technique to allow the formulation of predictions, or to test various properties of cABNs, although enumeration of concrete models or the identification of minimal networks is also supported. Thus, the enumeration of the entire set of minimal networks could be more expensive using our approach than with *caspo*. However, our method provides direct strategies for incorporating prior knowledge such as definite interactions that must always be included, or a requirement that each component must have an activator, and supports richer observations such as the cyclic behavior considered as part of the yeast cell cycle example. When certain constraints that are not easily incorporated as *caspo* problems are relaxed the two approaches lead to similar results, where small differences can be attributed to the richer Boolean updated functions considered in *caspo*, compared to set of regulation conditions we define.

## Model Summary and Compute Run Time

A summary of the models that we studied using RE:IN is provided in the table on the following page. Column headings are abbreviated as follows.

**C**: Number of components

**T.I.**: Total number of interactions

**P.I.**: Number of interactions marked as possible

**R.C.**: Number of regulation conditions per component

**C.M.**: Number of concrete models defined by the ABN, not reflecting the ‘activator required’ constraint

* No consistent models exist

** Only a single consistent model exists in terms of network topology

# Supplementary Figures


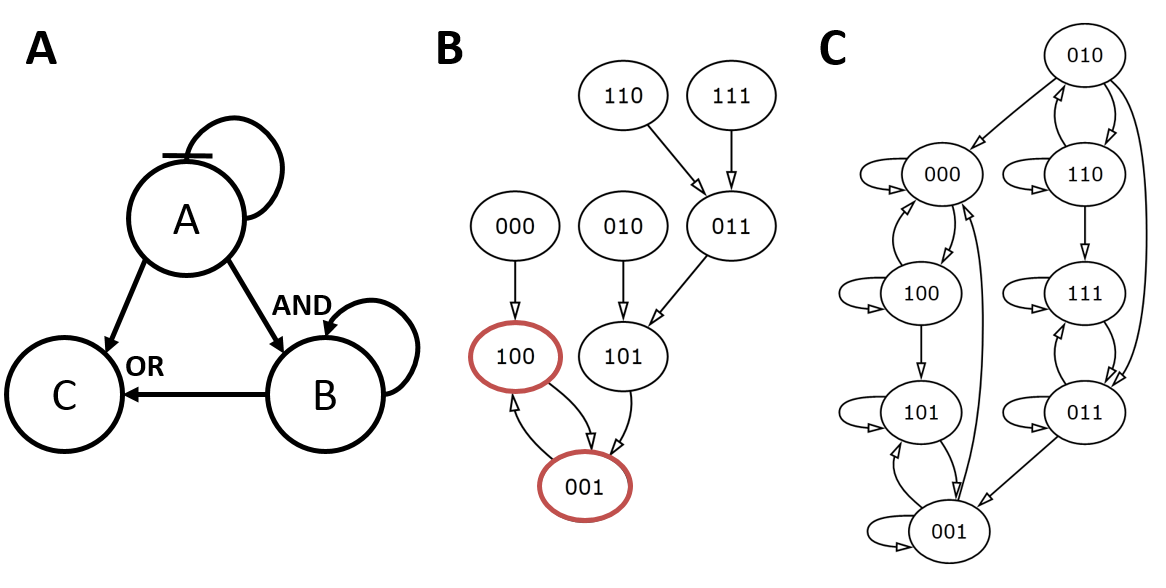


Figure S2: (**A**) Boolean network
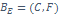
 with components
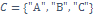
 and update functions
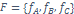
, where
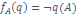
,
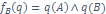
, and
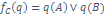
. (**B**) Transition system
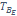
 constructed from
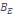
 by assuming synchronous updates, where the state label indicates the state of each species (e.g. for state
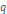
 labeled with 101 we have
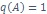
,
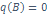
,
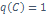
. All executions of this system converge to a two-state cycle, which is highlighted in red. (**C**) Transition system
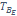
 constructed from
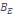
 by assuming asynchronous updates.


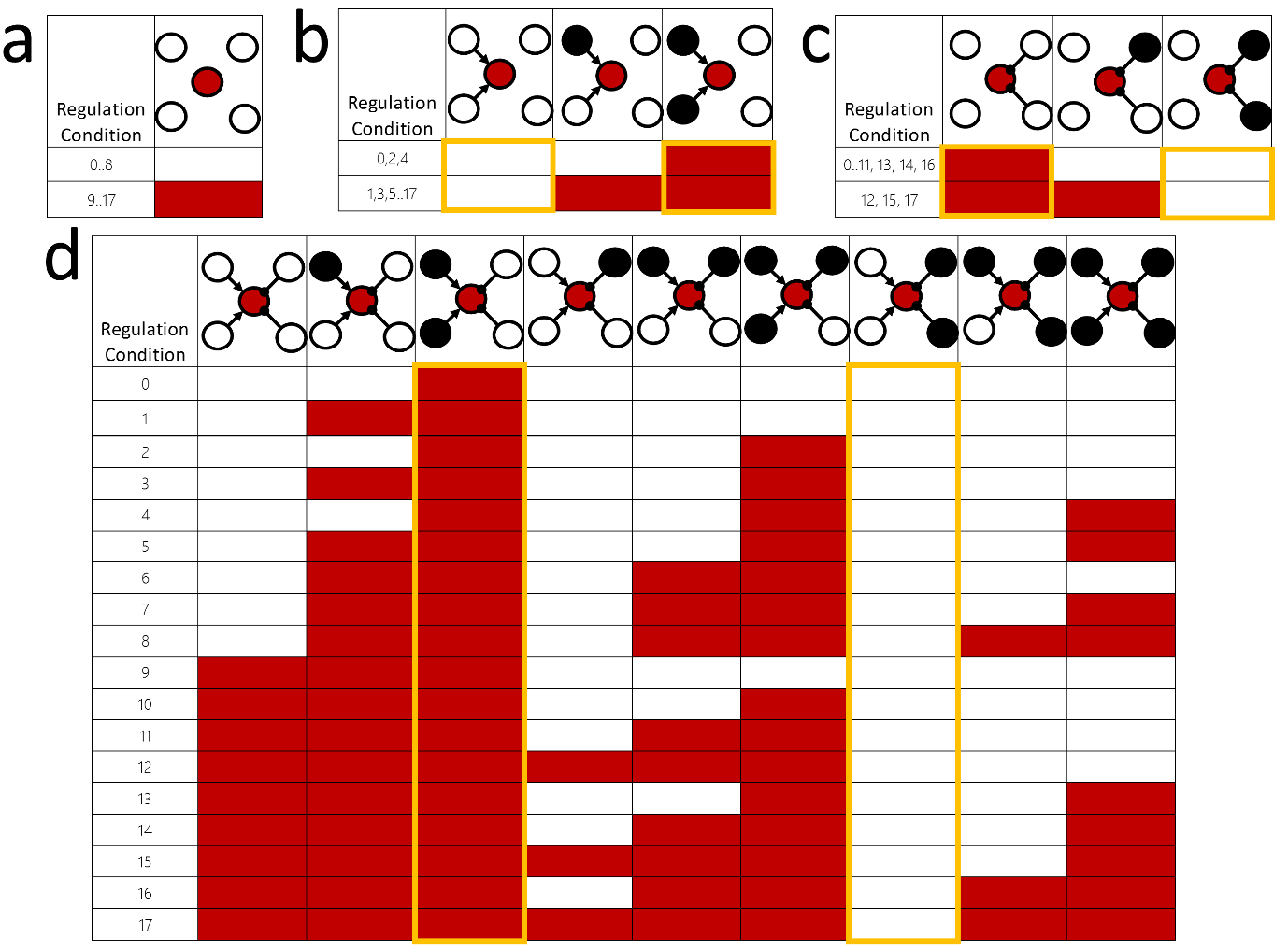


Figure S3: **The set of 18 regulation conditions (excluding the two threshold rules).** Each column represents a different context where the rule is applied. The updated state of the target node (shown in red in the header diagram) depends on the states of its regulators in the current state (represented as empty nodes if not available or filled nodes if available). Since we only consider the cases where none, some or all activators (repressors) of a target is available, it is sufficient to illustrate two regulators of each type. The cases where a component is non-inducible and non-repressible **(a)**, only inducible **(b)**, only repressible **(c)**, or both inducible and repressible **(d)**, are illustrated separately. The assumption that, for each regulation condition, the component cannot be constantly activated or repressed regardless of the state of each regulators is indicated by the constraints highlighted with yellow boxes.


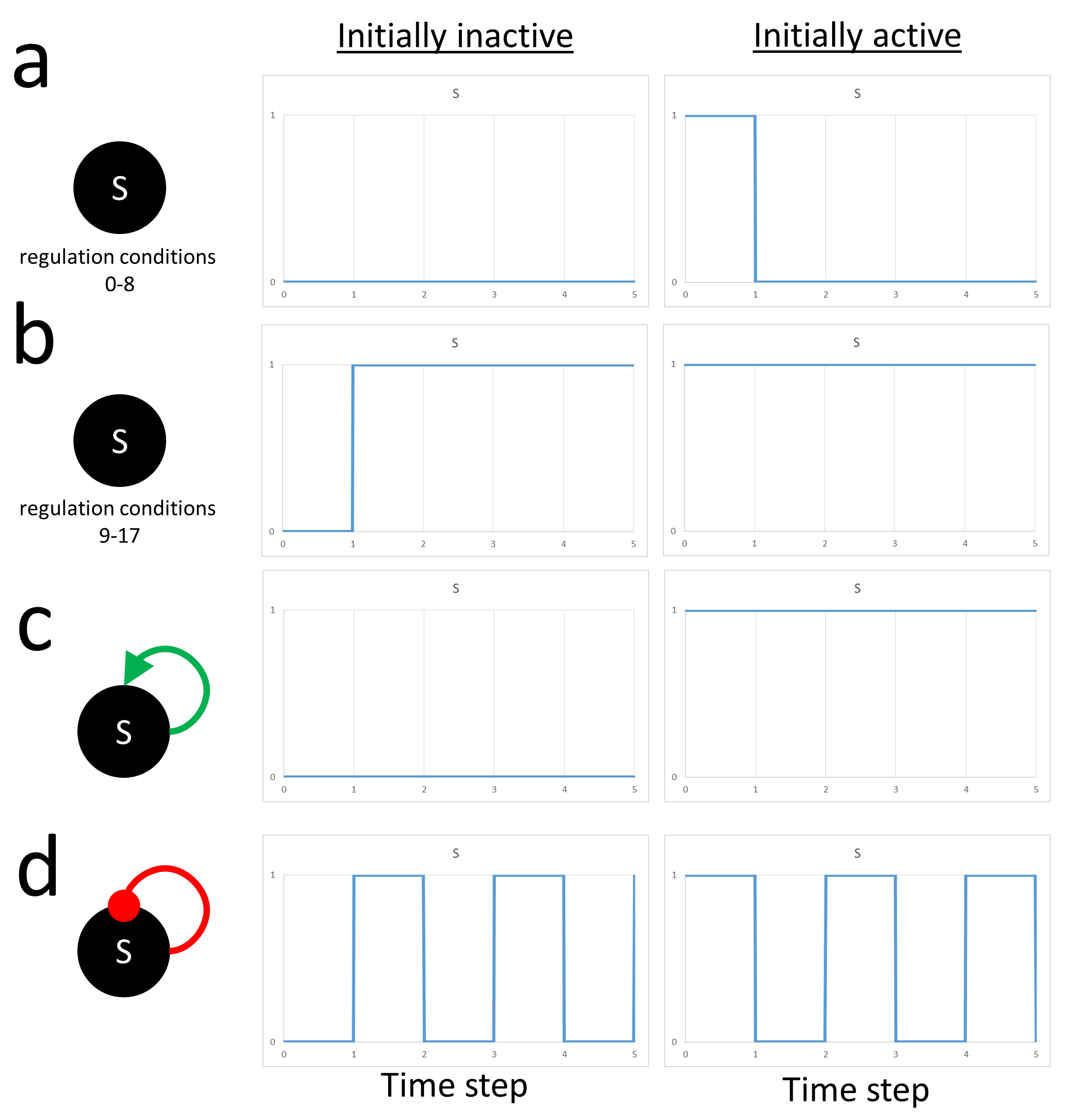


Figure S4: **Modelling of input signals with various dynamical behaviors**. Depending on the choice of regulation conditions (see Figure S3), a component without any regulators (i.e. an input signal) would be constantly inactive (regulation conditions 0 to 8) or active (regulation conditions 9 to 17). This property can be used to model self-activating signals that are inactive for a single time step and remain active through the rest of an experiment (first column of **b**) or vice versa for self-degrading signals (second column of **a**). A single self-activation ensures that the input signal is sustained throughout each experiment (either as active or inactive depending on the initial value) **c**, while a single self-repression can be used to model oscillating signals, **d**.

# References

Guziolowski, C., Videla, S., Eduati, F., Cokelaer, T., Siegel, A., Saez-rodriguez, J., Irisa, U.M.R., Beaulieu, C. De, 2013. Exhaustively characterizing feasible logic models of a signaling network using Answer Set Programming. Bioinformatics btt393.

Moignard, V., Macaulay, I.C., Swiers, G., Buettner, F., Schütte, J., Calero-Nieto, F.J., Kinston, S., Joshi, A., Hannah, R., Theis, F.J., Jacobsen, S.E., de Bruijn, M.F., Göttgens, B., 2013. Characterization of transcriptional networks in blood stem and progenitor cells using high-throughput single-cell gene expression analysis. Nat. Cell Biol. 15, 363–372. doi:10.1038/ncb2709

Moignard, V., Woodhouse, S., Haghverdi, L., Lilly, A.J., Tanaka, Y., Wilkinson, A.C., Buettner, F., Macaulay, I.C., Jawaid, W., Diamanti, E., Nishikawa, S.-I., Piterman, N., Kouskoff, V., Theis, F.J., Fisher, J., Göttgens, B., 2015. Decoding the regulatory network of early blood development from single-cell gene expression measurements. Nat. Biotechnol. 33, 269 – 276. doi:10.1038/nbt.3154

Muñoz Descalzo, S., Rué, P., Faunes, F., Hayward, P., Jakt, L.M., Balayo, T., Garcia-Ojalvo, J., Martinez Arias, A., 2013. A competitive protein interaction network buffers Oct4-mediated differentiation to promote pluripotency in embryonic stem cells. Mol. Syst. Biol. 9, 694. doi:10.1038/msb.2013.49

Xu, H., Ang, Y.-S., Sevilla, A., Lemischka, I.R., Ma’ayan, A., 2014. Construction and Validation of a Regulatory Network for Pluripotency and Self-Renewal of Mouse Embryonic Stem Cells. PLoS Comput. Biol. 10, e1003777. doi:10.1371/journal.pcbi.1003777
